# Supplementary material for: Dual apical methyltransferases orchestrate motility initiation in apicomplexan parasites
Source: Nat Commun. 2026 Aug 26;17:9068. doi: 10.1038/s41467-026-77078-y (PMC13518913; doi:10.1038/s41467-026-77078-y)
Supplement: Supplementary file 2 [file 41467_2026_77078_MOESM11_ESM.pdf]

nature portfolio

Peer Review File

Dual apical methyltransferases orchestrate motility initiation in apicomplexan parasites

Corresponding Author: Dr Elena Jimenez-Ruiz

Version 0:

Reviewer comments:

Reviewer #1

(Remarks to the Author)
Summary of the study
Qin et al. identify and characterize a previously unstudied SET-domain methyltransferase, PCKMT, as a key apical regulator of motility initiation in apicomplexan parasites. In Toxoplasma gondii tachyzoites, PCKMT localizes to the preconoidal rings and is essential for egress, invasion, and gliding motility, while having no detectable effect on intracellular replication. Conditional deletion of PCKMT abolishes conoid extrusion, prevents proper F-actin redistribution during egress, and mimics the loss of core motility components.
Using U-ExM, live and fixed high-resolution imaging, genetic perturbations, and complementation, the authors demonstrate that PCKMT acts upstream of the actin nucleator Formin-1 (FRM1): PCKMT is necessary to anchor FRM1 at the conoid, and a catalytically inactive PCKMT mutant cannot rescue FRM1 recruitment, conoid extrusion, or motility. Simultaneously, the apical methyltransferase AKMT is shown to be the main contributor to apical lysine methylation signals, with mostly distinct methylation and proximity-labeling signatures compared to PCKMT. The two enzymes localize independently at the apex but have a shared functional outcome: PCKMT/FRM1-dependent actin assembly is essential for AKMT's relocalization from the conoid to the cytosol during motility induction, while disruption of actin dynamics prevents AKMT redistribution.
Finally, the authors extend their findings to Plasmodium falciparum, showing that the PCKMT orthologue PfSET9 also localizes at the apex of merozoites, close to PfFRM1, and that conditional deletion of PfSET9 causes defective schizont-to-ring transition and decreased invasion, supporting the evolutionary conservation of this dual methyltransferase checkpoint across apicomplexans.
Overall assessment
This is an excellent and very impressive study. The work is beautifully executed, clearly written, and very well presented. The authors combine sophisticated quantitative cell biology (live and fixed high-resolution imaging, including U-ExM and STED), precise conditional genetics and complementation, and rigorous proteomic/methylome and TurboID analyses to define a dual apical methyltransferase module that enables motility initiation in apicomplexan parasites.
The discovery that PCKMT functions at the preconoidal rings to anchor FRM1 and thus facilitate actin-dependent conoid extrusion, AKMT relocalization, and motility adds a significant new layer to our understanding of how the apical complex integrates signaling and cytoskeletal remodeling. Extending these findings to P. falciparum through PfSET9 further highlights the evolutionary conservation and importance of this pathway. Overall, I see this study as a major advance in the field of apicomplexan cell biology.
I have only minor, presentation-focused suggestions that I believe could enhance clarity for readers.
Minor comments
1. Placement and framing of the Plasmodium data
The PfSET9 section is intriguing and offers a valuable hint of conservation, but the manuscript primarily focuses on T. gondii. You might consider (i) clearly framing this part as a brief "conservation" vignette in the text and/or (ii) moving some of the PfSET9 imaging (such as the U-ExM panels) to supplementary materials, with a more concise summary in the main figure. This could help keep the narrative centered on T. gondii while still highlighting the cross-species relevance.
2. Representation of P. falciparum developmental stages
For the Plasmodium knockout experiments, the stage-specific phenotype (schizont-to-ring transition failure) is key but could be visualized more clearly. I recommend re-plotting the blood-stage progression in stacked bar plots of stage proportions over time. This approach would help non-malaria experts better understand when and where the defect happens.
3. Clarifying the scope of the conservation claim
When discussing PfSET9, it may be helpful to clearly state that the data indicate an apical localization near PfFRM1 and an invasion defect consistent with the T. gondii phenotype, but a comprehensive mechanistic analysis similar to PCKMT/FRM1 has not yet been performed. A brief sentence to this effect would set expectations and emphasize that the Plasmodium section is mainly supportive rather than exhaustive.
4. Abstract: acronym definition

In the Abstract (e.g., line 33), when introducing PCKMT as “a preconoidal ring–associated lysine methyltransferase," consider visually indicating which words contribute to the acronym (for example, by underlining the relevant initial letters in Preconoidal, Conoid, K-methyltransferase) so that readers unfamiliar with the term can immediately see how it is derived.
5. Use of the term “checkpoint”
Around line 42, you describe this module as a “checkpoint.” The term “checkpoint” is often understood to imply a reversible decision point. If the process described here is not reversible in that sense, you might consider an alternative wording such as “regulatory module” or “regulatory step,” which may more accurately capture the biology.
6. Clarifying that PCKMT is essential for motility
In lines 80–81, you could explicitly state that PCKMT is “essential for motility” (in addition to egress/invasion) to clearly summarize the phenotypic impact of its deletion.
7. Definition of CGP and Pcr4
In lines 118–119, CGP and Pcr4 are introduced as acronyms without their full names. Please spell out the complete names on first mention to assist readers unfamiliar with these factors.
8. Apical ultrastructure in the PCKMT mutant
In line 122 and the associated figure, the larger space between the parasite plasma membrane and the conoid structure in the PCKMT mutant is intriguing. Could the authors comment on whether they can resolve what occupies this region in wild-type parasites and whether loss of actin filaments might alter membrane curvature or conoid positioning? Even a short speculative remark in the Discussion would be helpful.
9. Rationale for analysing blood-stage parasites
Starting from line 148, you transition to blood-stage P. falciparum, which lack a canonical conoid. It would be helpful to briefly mention the expression profile of SET9 across different life stages and to offer more rationale for concentrating on blood stages in this context. Additionally, you might note that ookinetes, which have a conoid and depend heavily on motility, could be an especially interesting stage to explore in future studies.
10. Nomenclature consistency for PfFRM1
In lines 154–155 and elsewhere, the text switches between “PfFRM1” and “FRM1.” I recommend consistently using “PfFRM1” when referring to the P. falciparum protein to prevent any confusion for readers.
Line 315: There is a different font for the word the letter “T” in the word “The”.
11. Methods clarifications
o Line 443: It would be helpful to include a brief description of how the PfSET9 mutant parasites were created (e.g., CRISPR-Cas9 strategy, targeted locus, selection scheme) and to add the PlasmoDB gene ID for SET9 for clarity.
o Line 740: Information on the anti-FORM1 antibody seems to be missing. Please include the source (commercial supplier or in-house production, along with any reference) and specify the dilution used.
o Supplementary Figure 3, panels D and E: Providing details about the DNA ladder used (brand, size range) would help in interpreting the band sizes in these gels.
Overall, these are minor points regarding wording and presentation. The core data are solid, the experiments are thorough, and the mechanistic model is convincing. I strongly support publication after the authors address these small suggestions.

Reviewer #2

(Remarks to the Author)
The phylum Apicomplexa comprises a diverse group of obligate intracellular parasites, including Toxoplasma gondii and Plasmodium falciparum. Central to their pathogenesis is a unique form of substrate-dependent locomotion known as gliding motility, a process driven by an actomyosin motor complex housed within the parasite's pellicle. While the structural components of this motor and the gross cytoskeletal architecture have been extensively mapped, the precise signaling mechanisms that translate upstream calcium flux into the mechanical initiation of motility have remained partially obscure. This report identifies a novel "dual methyltransferase" checkpoint that governs this critical transition. The authors synthesize data regarding a newly characterized enzyme, PCKMT (PreConoidal Ring-associated Lysine Methyltransferase), also identified in earlier structural studies as AKMT2, and its hierarchical relationship with the previously known effector AKMT (Apical Lysine Methyltransferase).

Through conditional genetic knockout, high-resolution cryo-electron tomography (cryo-ET), expansion microscopy (U-ExM), and proximity-labeling proteomics, the authors establish that PCKMT functions as a regulator of the conoid complex. Positioned at the preconoidal rings (PCRs), PCKMT recruits and anchors the actin nucleator Formin-1 (FRM1) in a catalytic-dependent manner. This anchoring event licenses the polymerization of apical F-actin, which is coupled to the release of the downstream methyltransferase AKMT and the subsequent recruitment of the glideosome-associated connector (GAC). The work integrates molecular findings with emerging atomic models of the invasion machinery, specifically elucidating the role of the unconventional myosin MyoL and its associated calmodulin-like light chains (CAM1 and CAM4) as probable calcium sensors that may activate this methylation cascade.

The presence of Tetratricopeptide Repeats (TPR) and Ankyrin repeats suggests that PCKMT acts as a scaffold itself, capable of bridging multiple protein complexes. The catalytic SET domain contains a conserved HxC motif (Histidine-Cysteine), which is the hallmark of active methyltransferases. PCKMT location is strictly dependent on the structural protein CGP. In CGP-null parasites, PCKMT fails to localize to the apex. However, depletion of downstream effectors, such as FRM1, does not displace PCKMT. Cryo-ET reconstructions of PCKMT-depleted mutants show that despite a loss of invasion capability, the conoid structure remains intact, proving that PCKMT is not a load-bearing component of the conoid. In its absence, conoid is structurally sound but functionally inert. The loss of PCKMT is lethal to the lytic cycle, comparable to the loss of core motor components such as actin or Myosin A.

PCKMT-depleted parasites exhibit a severe defect in induced conoid extrusion, mirroring FRM1 knockout and provide the first genetic link between the methyltransferase and the actin nucleation machinery. Without PCKMT, the parasite cannot position the actin-nucleating machinery. A catalytic-dead complementation strain of PCKMT failed to rescue the lytic cycle defects. Crucially, the mutant supported partial recruitment of FRM1. This result implies that PCKMT can weakly bind FRM1, but the methyltransferase activity is required to lock it in place or, more likely, to activate it. The study also identifies PfSET9 as an ortholog of PCKMT. While previous transcriptomic studies had detected set9 expression in sexual stages, its function in asexual blood stages was unknown. PfSET9 localizes to the apical tip of segmented merozoites, colocalizing with PfFRM1. Conditional excision of set9 causes a lethal block in the transition from schizonts (replication) to rings (new infection), mirroring the invasion defect in the TgPCKMT-KO strain. The methylation checkpoint for actin assembly thus appears to be a conserved feature of apicomplexan biology. Conceptually, this report demonstrates that lysine methylation acts not merely as an epigenetic marker but as a rapid, reversible post-translational switch controlling the biophysics of parasite invasion.

Major Revision

(1) To provide a truly comprehensive understanding, authors must integrate their findings with the atomic structural models of the conoid recently published by Zeng et al. (PMID: 41366525). Zeng et al. solved the structure of the Preconoidal Ring 2 (PCR-P2) and identified a core complex of proteins including AKMT2 (a.k.a. PCKMT in this report), MyoL, CAM1 & CAM4 and MLC4. This work describes how calcium triggers motility, focusing on the downstream methyltransferases. The Zeng et al. structure provides the upstream sensor. Can authors comment if the MyoL-CAM1/4 complex acts as the resident calcium sensor of the PCRs? In low calcium (resting), MyoL maintains a conformation that obscures PCKMT or keeps FRM1 in an inhibited state. Calcium influx leads to its binding to the EF-hands of CAM1 and CAM4, inducing a structural rearrangement of the MyoL neck region. This mechanical shift exposes the PCKMT active site or facilitates the stable docking of FRM1. MyoL deletion also phenocopies the PCKMT knockout. Did the authors find MyoL and CAMs in the proximity labeling? Given that PCKMT (AKMT2) and MyoL are atomic neighbors in the PCR-P2 ring, MyoL peptides should likely be present in the PCKMT-TurboID dataset. The integration of two articles creates a unified model where MyoL serves as the sensor, and PCKMT functions as the effector.

(2) In PMID: 41366525, the authors report the localization of seven identified P2 essential proteins: FRM1, FLM1, FLM2, AKMT2 (PCKMT), PCR10, CAM4, and SEC24. This study also positions MyoL as part of the essential proteins in PCR-P2. The deletion of MyoL severely impaired parasite growth and invasion. Furthermore, the authors propose that the MyoL neck region interacts with three potential myosin light chain subunits (CAM1, CAM4, and MLC4) that contain calcium-binding EF-hand domains. Did the authors find these proteins in proximity labeling assays, and if so, would calcium signaling in this region be an additional step in the mechanism that leads to conoid extrusion? In this same context. Determining the role of calcium in motility initiation will strengthen the manuscript, given the critical role of calcium in this process, as demonstrated by the use of calcium ionophores. Furthermore, the elimination of MyoL may influence PCKMT activity during the initiation of motility.

(3) A major limitation is the identity of the directly methylated substrate. FRM1 is the most logical candidate. Methylation could block an autoinhibitory interaction or create a binding surface for the conoid scaffold. The report notes that actin itself was not enriched in methyl-peptide pulldowns, suggesting the regulation is at the level of the nucleator (FRM1) rather than the filament itself. Given the proximity, methylation of MyoL could stabilize the open conformation of the calcium sensor. Future experiments focusing specifically on the PCR-P2 complex components are required to identify the precise lysine residue(s) that act as the molecular switch.

(4) The authors should integrate the following information in the results and discussion sections, where appropriate (perhaps the flow of information in the manuscript can be more coherent). PCKMT is stably associated with the PCRs. Its interactome (TurboID) is enriched for apical proteins, but surprisingly, it lacks many cytoskeletal motors, reflecting its specific role as a regulator rather than a motor component. AKMT is dynamically associated with the conoid. Its interactome is rich in myosin complex components, vesicle organization factors, and cytoskeletal proteins. This reflects its broader role in remodeling the pellicle during translocation. In PCKMT-depleted parasites, AKMT localization is aberrant. In resting, AKMT localizes to the conoid normally. Upon calcium stimulation, AKMT fails to release into the cytosol. This establishes the directionality of the cascade: PCKMT is upstream of AKMT. The release of AKMT appears not to be a direct response to calcium but is dependent on the actin polymerization licensed by PCKMT. Fluorescence Recovery After Photobleaching (FRAP) analysis highlights the dynamic nature of these proteins. GAC-YFP exhibits rapid recovery (12.8s), indicating a highly mobile pool that constantly exchanges with the cytosol. AKMT-YFP shows slower but significant recovery (42.3s). IMC1-Halo shows no recovery (structural control). The dynamic exchange of AKMT and GAC, even in the resting state, suggests the system is "primed." They are not cemented in place but are held in a dynamic equilibrium, ready to be biased towards translocation by the explosive polymerization of actin triggered by PCKMT.

(5) In wild-type parasites, apical actin fluorescence (CbHalo) is high, while in Cytochalasin D (CytD) treated parasites, it is low. The same assay for the catalytically dead (PCKMT-H542A) mutant can prove that the catalytic defect leads to a polymerization defect. Could the authors repeat the experiment using the same strain and determine the role of methyltransferase activity in actin polymerization and the initiation of motility?

Minor

(1) The manuscript should include the DiCre\Delta Ku80 parental control data in the invasion assay graph (Fig. 1i) to allow for direct normalization against the plaque and gliding assays.

(2) Add a label to Figure 6a stating that it represents the condition with the ionophore so that it is not confused with Figure 4c, where the localization of AKMT is only observed in the conoid. The ambiguity in AKMT localization must be resolved by explicitly labeling panels as "+ Calcium Ionophore" versus "Resting."

(3) The quantification in Figure 6e should be refined to report "Cytosolic/Total Fluorescence Ratio" rather than a binary "Translocated" score, to better capture the nuance of partial defects in mutants.

(4) Strict adherence to strain labels (e.g., loxPpckmt-Halo vs. DiCre) in figure legends is essential to avoid confusion between wild-type and non-induced controls. Please include the plaque number quantification in the supplementary material. Figure 2 b and c: Please label the strains used.

(5) To maintain consistency in the figures, the authors could place the boxes indicating the magnification in all the figures; for example, they are indicated in Figure 1d, but not in Figure 1e.

(6) Line 304, Figure 6b should be in the place of Figure 6a.

(7) Figure 6e is missing the y-axis label.

(8) Line 309, Figure 6b, should be Figure 6c. Please change accordingly.

(9) Line 312, Figure 6c should be 6d.

(10) Lines 319-322: The quantification they performed is not clear. The images in Figures 6a and 6 b show AKMT in both the apical region and the cytosol; however, this is not reflected in the quantification in Figure 6e.

Reviewer #3

(Remarks to the Author)
Summary
This study reports on a novel methyl transferase (PCKM1) identified in T gondii, with a similar function in P. falciparum. Localization of PCKM1 at the pre-conoidal ring is largely independent from other know PCR proteins, whereas only FRM1 localization to the conoid appears to be dependent on PCKM1 activity, and there is no effect on the stability of the cytoskeleton. Hence, a very stringent 1 on 1 relationship, and it is remarkable that this so nicely came together here (wide methylome and TurboID nets were cast). I appreciated the detail that FRM1 is normally recruited to the PCR during budding, but that PCKM1 causes a maturation to retain FRM1 in mature parasites. Mechanistically, the model presented in Fig 7 says is all: PCKM1 recruits FRM1 to promote conoid extrusion during parasite egress (downstream of Ca and cGMP kinases), which in turn is required to start actin polymerization needed for gliding motility through GAC and AKMT translocation from the conoid to cytoplasm. Although new questions arise as nicely laid out in the discussion, this study is a major mechanistic leap for the field. Overall, this is a very well-designed study including all controls and quantitative assessments where possible. I am on board with result interpretations and assembled model. However, I have many relatively minor remarks on how data are presented or are collected

Specific points
1. Please provide the ToxoDB GeneID in the main manuscript (finally found in Table S3).
2. Many legends do not explain the size of the scalebar shown. Please add throughout all panels/legends.
3. Fig S2a. An important detail is not explained very well in the manuscript. Looks like these parasites are undergoing budding, as 2 ICAP16 and PCKMT spots in each parasite are seen that are not in conoid, which I assume are daughters. Unfortunately, TLAP1 does not report on the presence of daughter buds. Digging in the literature, I could not find any details on ICAP16 in assembling daughters/conoids. Hence, some additional explanation or exploration in daughter assembly would be very welcome here (e.g. using an early daughter bud reporter like ISP1, or centrosome marker which duplicates before budding starts). Reading further, Fig 3f covers this aspect somewhat, but not in the ICAP16 context...
4. Fig S2b-e. It would be helpful to add the parasite strain in the figure itself (now it is in the legend). Video 3 is properly labeled, so apply the same here.
5. Fig 1h. These plaque assays are not the clearest image as it is hard to make out plaques, and the difference with +Rapa LoxPpckmt is very subtle (e.g. I see 2 clearly white spots of different size in Dicre -Rapa, but I do not think those are plaques?). Please replace with better stained plaque assay. Fig 3a is marginally better, but could also benefit from clearer panels
6. Fig 2b-d. Please add the color-key for factors visualized in the figure panels rather than the legend. Panel 2d legend does not explain what blue and magenta represent at all (the blue looks like the moving junction???). Colors are also not explained for Fig S6b and c.
7. Line 164-166. This sentence would be better as an opener in the next section. A stylistic opinion?
8. Fig S5a. I am trying to understand what exactly was done here. the number of the PCRs, do they represent the order? “5. Insertion PCR” only has 1, slanted arrow, which I am not sure at all how to interpret? I thought this construct was targeted into the UPRT locus, but the pGEM-T flanks seem to suggest otherwise?? Or is this a Plasmodium schematic?
9. Fig S6b,c. Please explain in the legend what the white arrows point at.
10. Fig S8c,d legends. These western blots do not permit the conclusion that in c apical and cytosolic proteins are biotinylated, and in d preconoidal and apical apicomplex-associated proteins. Please revise legends (e.g. remove these comments, or refer to Fig 5 corresponding BioID panels).

11. Fig 5d, f, h. Please add in the panel whether methylome or biotinylation is plotted.
12. Fig 10b. TGGT1_212780 was described as SFA5 (https://doi.org/10.1186/s44280-024-00043-1); ASAF1 was assigned SFA4 in the same paper. Looks like SFA5 might indeed be an ASF component, but that ASAF1 is not confirmed on the SFA?
13. Fig 6e has no y-axis label. It is not immediately clear how AKMT translocation was quantified – signal intensity or an arbitrary score? The source data suggests and arbitrary score but the description there is not clear either. Please clarify
14. Line 329 refers to Fig 6d,e. which should be 6e,f. Movie S7 was not playing for me (all others did), but Fig e,f are very clear.

Reviewer #4

(Remarks to the Author)
I co-reviewed this manuscript with one of the reviewers who provided the listed reports. This is part of the Nature Communications initiative to facilitate training in peer review and to provide appropriate recognition for Early Career Researchers who co-review manuscripts.

Author Rebuttal letter:

RESPONSE TO REVIEWER COMMENTS
We thank the reviewers for their careful evaluation of our manuscript and for the constructive and insightful comments. We are pleased that the reviewers found the study to be of high quality and of significant interest, and we appreciate their positive assessment of the conceptual advance and experimental rigor. We believe that the revisions made in response to their suggestions have strengthened the clarity, presentation, and overall impact of the manuscript.
Below, we provide a point-by-point response to all reviewer comments. Reviewer comments are shown in black, and our responses are indicated in blue font.

Reviewer #1:
Summary of the study
Qin et al. identify and characterize a previously unstudied SET-domain methyltransferase, PCKMT, as a key apical regulator of motility initiation in apicomplexan parasites. In Toxoplasma gondii tachyzoites, PCKMT localizes to the preconoidal rings and is essential for egress, invasion, and gliding motility, while having no detectable effect on intracellular replication. Conditional deletion of PCKMT abolishes conoid extrusion, prevents proper F-actin redistribution during egress, and mimics the loss of core motility components.
Using U-ExM, live and fixed high-resolution imaging, genetic perturbations, and complementation, the authors demonstrate that PCKMT acts upstream of the actin nucleator Formin-1 (FRM1): PCKMT is necessary to anchor FRM1 at the conoid, and a catalytically inactive PCKMT mutant cannot rescue FRM1 recruitment, conoid extrusion, or motility. Simultaneously, the apical methyltransferase AKMT is shown to be the main contributor to apical lysine methylation signals, with mostly distinct methylation and proximity-labeling signatures compared to PCKMT. The two enzymes localize independently at the apex but have a shared functional outcome: PCKMT/FRM1-dependent actin assembly is essential for AKMT's relocalization from the conoid to the cytosol during motility induction, while disruption of actin dynamics prevents AKMT redistribution.
Finally, the authors extend their findings to Plasmodium falciparum, showing that the PCKMT orthologue PfSET9 also localizes at the apex of merozoites, close to PfFRM1, and that conditional deletion of PfSET9 causes defective schizont-to-ring transition and decreased invasion, supporting the evolutionary conservation of this dual methyltransferase checkpoint across apicomplexans.
Overall assessment
This is an excellent and very impressive study. The work is beautifully executed, clearly written, and very well presented. The authors combine sophisticated quantitative cell biology (live and fixed high-resolution imaging, including U-ExM and STED), precise conditional genetics and complementation, and rigorous proteomic/methylome and TurboID analyses to define a dual apical methyltransferase module that enables motility initiation in apicomplexan parasites.
The discovery that PCKMT functions at the preconoidal rings to anchor FRM1 and thus facilitate actin-dependent conoid extrusion, AKMT relocalization, and motility adds a significant new layer to our understanding of how the apical complex integrates signaling and cytoskeletal remodeling. Extending these findings to P. falciparum through PfSET9 further highlights the evolutionary conservation and importance of this pathway. Overall, I see this study as a major advance in the field of apicomplexan cell biology.

I have only minor, presentation-focused suggestions that I believe could enhance clarity for readers.
We thank Reviewer #1 for this thoughtful and generous assessment of our work. We greatly appreciate their recognition of both the technical rigor and the conceptual advance of the study. We have addressed all points raised below and believe that these revisions have further strengthened the manuscript.
Minor comments
1. Placement and framing of the Plasmodium data
The PfSET9 section is intriguing and offers a valuable hint of conservation, but the manuscript primarily focuses on T. gondii. You might consider (i) clearly framing this part as a brief “conservation” vignette in the text and/or (ii) moving some of the PfSET9 imaging (such as the U-ExM panels) to supplementary materials, with a more concise summary in the main figure. This could help keep the narrative centered on T. gondii while still highlighting the cross-species relevance.
We appreciate the reviewer’s suggestion and have modified the data presented in the main figure for the Plasmodium dataset. We now include the localization of PfSET9 relative to PfFRM1, as well as growth curves and the developmental stages requested in the point below. As these data are somewhat preliminary and more extensive experiments with this parasite are still needed, we have kept the focus on this aspect to a minimum. We moved the rest of the panels to supplementary information as suggested.
2. Representation of P. falciparum developmental stages
For the Plasmodium knockout experiments, the stage-specific phenotype (schizont-to-ring transition failure) is key but could be visualized more clearly. I recommend re-plotting the blood-stage progression in stacked bar plots of stage proportions over time. This approach would help non-malaria experts better understand when and where the defect happens.
We thank the reviewer for the suggestion to visualise stage progression. We have now included a bar graph to provide a clearer representation of parasite fate during progression (Fig. 2h). Because the synchronization and inductions had to be repeated, we also repeated the growth curves, which reproduced the same pattern observed in the previous experiment (now Fig. 2g). This graph has been updated accordingly.
In our tightly synchronised cultures, we observed disappearance of schizonts without accompanying ring formation upon PfSET9 depletion. This might indicate a primary effect on invasion and potentially in part egress. Strikingly, we observed the appearance of gametocyte-like parasites in the induced cultures. Although these parasites were present at very low numbers, they were not observed under non-induced conditions. This observation suggests that PfSET9 may have a dual function and could be involved in the early stages of gametocytogenesis, as proposed by Brancucci and colleagues1. However, since further studies are needed to confirm this effect in other P. falciparum strains that are more amenable to in vitro gametocytogenesis, we remain cautious in interpreting these findings. Overall, the phenotype primarily reflects a block in invasion rather than in erythrocytic development, which we have now clarified in the text.
“We next quantified the parasite stages at each time point post-induction and observed a marked reduction in rings, despite schizont numbers remaining comparable to those in non-induced parasites, ultimately leading to a severe reduction in parasites in the subsequent cycle (Fig. 2h). These findings indicate a failure in invasion, and potentially egress, in parasites depleted of PfSET9. Intriguingly, early gametocytes were observed in induced parasites (Fig. 2h; Supplementary Fig. 3i), raising the possibility of an association with gametocyte formation.“ (Page 7, lines 139-145)
3. Clarifying the scope of the conservation claim
When discussing PfSET9, it may be helpful to clearly state that the data indicate an apical localization near PfFRM1 and an invasion defect consistent with the T. gondii phenotype, but a comprehensive mechanistic analysis similar to PCKMT/FRM1 has not yet been performed. A brief sentence to this effect would set expectations and emphasize that the Plasmodium section is mainly supportive rather than exhaustive.
We now include a clarification in the results section:
“Together, these data identify TgPCKMT as an apical lysine methyltransferase that is essential for conoid-dependent motility in T. gondii and point to an evolutionarily conserved role for its Plasmodium orthologue, PfSET9, in invasion-associated processes across apicomplexans. Our data support an apical localisation of PfSET9 in proximity to PfFRM1 and reveal an invasion defect reminiscent of that observed in T. gondii. However, a detailed mechanistic investigation of PfSET9 function, comparable to that performed for TgPCKMT/TgFRM1, remains the subject of ongoing work.“(see page 7 lines 146-152)

4. Abstract: acronym definition
In the Abstract (e.g., line 33), when introducing PCKMT as “a preconoidal ring–associated lysine methyltransferase," consider visually indicating which words contribute to the acronym (for example, by underlining the relevant initial letters in Preconoidal, Conoid, K-methyltransferase) so that readers unfamiliar with the term can immediately see how it is derived.
We have revised the Abstract to visually indicate the words contributing to the PCKMT acronym when it is first introduced, as suggested.
5. Use of the term “checkpoint”
Around line 42, you describe this module as a “checkpoint.” The term “checkpoint” is often understood to imply a reversible decision point. If the process described here is not reversible in that sense, you might consider an alternative wording such as “regulatory module” or “regulatory step,” which may more accurately capture the biology.
We agree with the reviewer and have replaced the term “checkpoint” with “regulatory module” in the Abstract and throughout the manuscript where appropriate, to better reflect the ordered but not necessarily reversible nature of the process described.
6. Clarifying that PCKMT is essential for motility
In lines 80–81, you could explicitly state that PCKMT is “essential for motility” (in addition to egress/invasion) to clearly summarize the phenotypic impact of its deletion.
We have revised the Introduction to explicitly state that PCKMT is essential for parasite motility, in addition to its roles in invasion and egress (see page 4 lines 51-52).
7. Definition of CGP and Pcr4
In lines 118–119, CGP and Pcr4 are introduced as acronyms without their full names. Please spell out the complete names on first mention to assist readers unfamiliar with hese factors.
We have included definitions for the acronyms CGP and Pcr4 at their first mention in the manuscript (lines 25 and 94, respectively).
8. Apical ultrastructure in the PCKMT mutant
In line 122 and the associated figure, the larger space between the parasite plasma membrane and the conoid structure in the PCKMT mutant is intriguing. Could the authors comment on whether they can resolve what occupies this region in wild-type parasites and whether loss of actin filaments might alter membrane curvature or conoid positioning? Even a short speculative remark in the Discussion would be helpful.
We thank the reviewer for highlighting this observation. In cryo-electron tomography analyses, we occasionally observed an increased spacing between the parasite plasma membrane and the conoid in PCKMT-depleted parasites. However, due to the limited sampling inherent to cryo-ET and the absence of consistent membrane abnormalities in fluorescence or expansion microscopy (see image below), we cannot determine whether this reflects a genuine consequence of impaired actin polymerisation or represents a preparation-related artefact. It is nevertheless tempting to speculate that local actin, and glideosome assembly, may contribute to maintaining membrane curvature during motility initiation, a possibility that will require targeted investigation in future studies.
We include here images of parasite membrane using U-ExM marking the membrane and subpellicular microtubules for the reviewers (response figure 1). We have decided not to include this image in the manuscript since it does not add meaningful data to this work.
loxPpckmt-Halo
Merge α- AcTub α- Toxoplasma Zoom Response figure 1:
d in Representative U-ExM images
acquired on a Leica DMi8 widefield
microscope showing the spatial
relationship between the plasma
membrane and the conoid in
-Rapa

untreated and rapamycin-treated
(PCKMT-depleted) parasites.
Variable spacing between the
plasma membrane and the conoid
was observed in both conditions,
including examples of closely
apposed and more detached
morphologies. These observations

indicate that plasma membrane–conoid distance can vary independently of PCKMT depletion, +Rapa

suggesting that this feature may be influenced by preparation and/or expansion-related effects. Overview images are shown with a 5 µm scale bar; zoomed views are shown with a 2 µm scale bar.

9. Rationale for analysing blood-stage parasites
Starting from line 148, you transition to blood-stage P. falciparum, which lack a canonical conoid. It would be helpful to briefly mention the expression profile of SET9 across different life stages and to offer more rationale for concentrating on blood stages in this context. Additionally, you might note that ookinetes, which have a conoid and depend heavily on motility, could be an especially interesting stage to explore in future studies.
We thank the reviewer for this helpful suggestion. According to transcription data in PlasmoDB, PfSET9 is also expressed during asexual blood stages, with peak expression in the schizont stage. Although blood-stage Plasmodium parasites have long been considered to lack a canonical conoid, recent ultrastructure expansion microscopy studies have revealed previously unappreciated apical ring-like structures in merozoites, suggesting a more complex apical organisation than previously recognized2. While we cannot exclude the possibility that PfSET9 may fulfil additional or distinct functions during blood-stage development, its apical localisation in close proximity to PfFRM1 and the pronounced invasion defect upon depletion strongly support a role in invasion-associated processes. We now also note that ookinetes, which possess a more prominent conoid and rely heavily on motility, represent particularly interesting stages for future investigation, but were beyond the scope of the present study. We include now in the discussion:
"Given the reduced conoid complex of Plasmodium parasites2, these findings raise questions about how PfSET9 is positioned within this simplified apical architecture and whether it contributes to motility across the life cycle. In particular, ookinetes, which harbour a more prominent conoid and rely heavily on motility, represent attractive systems to further explore PfSET9 function in future studies." (Page 18-19 lines 448-453)
10. Nomenclature consistency for PfFRM1
In lines 154–155 and elsewhere, the text switches between "PfFRM1" and "FRM1." I recommend consistently using "PfFRM1" when referring to the P. falciparum protein to prevent any confusion for readers.
To avoid confusion, we have revised the manuscript to consistently distinguish between T. gondii and P. falciparum proteins and now use species-specific nomenclature throughout (e.g., PfFRM1 for the P. falciparum protein or TgFRM1 for T. gondii).
Line 315: There is a different font for the word the letter "T" in the word "The".
This typographical issue has been corrected.
11. Methods clarifications
o Line 443: It would be helpful to include a brief description of how the PfSET9 mutant parasites were created (e.g., CRISPR-Cas9 strategy, targeted locus, selection scheme) and to add the PlasmoDB gene ID for SET9 for clarity.
We expanded the section about the generation of the PfSET9 conditional mutant and moved the Plasmodium methodology to align with the Toxoplasma methods. We also added the corresponding PlasmoDB gene ID at the relevant point in the Methods. (See pages 19-23)
o Line 740: Information on the anti-FRM1 antibody seems to be missing. Please include the source (commercial supplier or in-house production, along with any reference) and specify the dilution used.
We have added the source and dilution of the anti-FRM1 antibody to the Immunofluorescence Assay (IFA) Methods section for Plasmodium. (See page 23, line 567)
o Supplementary Figure 3, panels D and E: Providing details about the DNA ladder used (brand, size range) would help in interpreting the band sizes in these gels.
We have now labelled the bands of the ladder for clearer interpretation of our PCR

gels.
Overall, these are minor points regarding wording and presentation. The core data are solid, the experiments are thorough, and the mechanistic model is convincing. I strongly support publication after the authors address these small suggestions.

Reviewer #2 :
The phylum Apicomplexa comprises a diverse group of obligate intracellular parasites, including Toxoplasma gondii and Plasmodium falciparum. Central to their pathogenesis is a unique form of substrate-dependent locomotion known as gliding motility, a process driven by an actomyosin motor complex housed within the parasite's pellicle. While the structural components of this motor and the gross cytoskeletal architecture have been extensively mapped, the precise signaling mechanisms that translate upstream calcium flux into the mechanical initiation of motility have remained partially obscure. This report identifies a novel "dual methyltransferase" checkpoint that governs this critical transition. The authors synthesize data regarding a newly characterized enzyme, PCKMT (PreConoidal Ring-associated Lysine Methyltransferase), also identified in earlier structural studies as AKMT2, and its hierarchical relationship with the previously known effector AKMT (Apical Lysine Methyltransferase).
Through conditional genetic knockout, high-resolution cryo-electron tomography (cryo-ET), expansion microscopy (U-ExM), and proximity-labeling proteomics, the authors establish that PCKMT functions as a regulator of the conoid complex. Positioned at the preconoidal rings (PCRs), PCKMT recruits and anchors the actin nucleator Formin-1 (FRM1) in a catalytic-dependent manner. This anchoring event licenses the polymerization of apical F-actin, which is coupled to the release of the downstream methyltransferase AKMT and the subsequent recruitment of the glideosome-associated connector (GAC). The work integrates molecular findings with emerging atomic models of the invasion machinery, specifically elucidating the role of the unconventional myosin MyoL and its associated calmodulin-like light chains (CAM1 and CAM4) as probable calcium sensors that may activate this methylation cascade. The presence of Tetratricopeptide Repeats (TPR) and Ankyrin repeats suggests that PCKMT acts as a scaffold itself, capable of bridging multiple protein complexes. The catalytic SET domain contains a conserved HxC motif (Histidine-Cysteine), which is the hallmark of active methyltransferases. PCKMT location is strictly dependent on the structural protein CGP. In CGP-null parasites, PCKMT fails to localize to the apex. However, depletion of downstream effectors, such as FRM1, does not displace PCKMT. Cryo-ET reconstructions of PCKMT-depleted mutants show that despite a loss of invasion capability, the conoid structure remains intact, proving that PCKMT is not a load-bearing component of the conoid. In its absence, conoid is structurally sound but functionally inert. The loss of PCKMT is lethal to the lytic cycle, comparable to the loss of core motor components such as actin or Myosin A.
PCKMT-depleted parasites exhibit a severe defect in induced conoid extrusion, mirroring FRM1 knockout and provide the first genetic link between the methyltransferase and the actin nucleation machinery. Without PCKMT, the parasite cannot position the actin-nucleating machinery. A catalytic-dead complementation strain of PCKMT failed to rescue the lytic cycle defects. Crucially, the mutant supported partial recruitment of FRM1. This result implies that PCKMT can weakly bind FRM1, but the methyltransferase activity is required to lock it in place or, more likely, to activate it. The study also identifies PfSET9 as an ortholog of PCKMT. While previous transcriptomic studies had detected set9 expression in sexual stages, its function in asexual blood stages was unknown. PfSET9 localizes to the apical tip of segmented merozoites, colocalizing with PfFRM1. Conditional excision of set9 causes a lethal block in the transition from schizonts (replication) to rings (new infection), mirroring the invasion defect in the TgPCKMT-KO strain. The methylation checkpoint for actin assembly thus appears to be a conserved feature of apicomplexan biology.
Conceptually, this report demonstrates that lysine methylation acts not merely as an epigenetic marker but as a rapid, reversible post-translational switch controlling the biophysics of parasite invasion.
We thank Reviewer #2 for this exceptionally thoughtful and integrative assessment of our work, and for placing our findings within the broader context of emerging structural and mechanistic models of motility initiation. We particularly appreciate the reviewer's synthesis of our data with recent advances in apical complex architecture and calcium-responsive regulation.
We would like to clarify that PCKMT was originally identified in our previous work as a CGP-interacting protein at the preconoidal rings (Li et al., 20253), and the name PreConoidal Lysine MethylTransferase (PCKMT) was deliberately chosen to reflect its highly restricted and stable localisation to this structure. In contrast, AKMT represents a more broadly apical methyltransferase that is not fixed to a single architectural

element, consistent with its dynamic behaviour revealed by FRAP analyses in the present study.
We note that the structural studies cited by the reviewer (Zeng et al., 20254) and its companion work on FRM1 (Qian et al., 20255) were released after submission of our manuscript. We are pleased to now integrate our findings with these highly complementary studies from the Sibley and Zhang laboratories, which together provide a convergent view of the PCR-P2 region as a regulatory hub integrating calcium sensing, cytoskeletal organisation, and enzymatic control of motility initiation.
Major Revision
(1) To provide a truly comprehensive understanding, authors must integrate their findings with the atomic structural models of the conoid recently published by Zeng et al. (PMID: 41366525). Zeng et al. solved the structure of the Preconoidal Ring 2 (PCR-P2) and identified a core complex of proteins including AKMT2 (a.k.a. PCKMT in this report), MyoL, CAM1 & CAM4 and MLC4. This work describes how calcium triggers motility, focusing on the downstream methyltransferases. The Zeng et al. structure provides the upstream sensor. Can authors comment if the MyoL-CAM1/4 complex acts as the resident calcium sensor of the PCRs? In low calcium (resting), MyoL maintains a conformation that obscures PCKMT or keeps FRM1 in an inhibited state. Calcium influx leads to its binding to the EF-hands of CAM1 and CAM4, inducing a structural rearrangement of the MyoL neck region. This mechanical shift exposes the PCKMT active site or facilitates the stable docking of FRM1. MyoL deletion also phenocopies the PCKMT knockout. Did the authors find MyoL and CAMs in the proximity labeling? Given that PCKMT (AKMT2) and MyoL are atomic neighbors in the PCR-P2 ring, MyoL peptides should likely be present in the PCKMT-TurboID dataset. The integration of two articles creates a unified model where MyoL serves as the sensor, and PCKMT functions as the effector.
We thank the reviewer for highlighting the importance of integrating our findings with the recent atomic model of the PCR-P2 ring by Zeng et al. We have revised the manuscript to better place our data within this structural framework.
The study by Zeng et al4. reveals that TgPCKMT (referred to as AKMT2), TgMyoL, TgFRM1 and calmodulin-like light chains are arranged in close spatial proximity within the PCR-P2 complex, supporting the existence of a highly organised regulatory platform at the conoid. In this context, the TgMyoL–CAM complex is well positioned to act as a calcium-responsive structural module, and could in principle function upstream of, or in coordination with, the TgPCKMT–TgFRM1 axis.
Consistent with this organisation, TgMyoL was detected in the TgPCKMT proximity-labelling dataset and was also enriched in the TgAKMT methylome dataset, supporting a close spatial association between TgMyoL and the apical methyltransferase network. To further explore the relationship between these components, we examined TgPCKMT-Halo localisation upon TgMyoL depletion. TgPCKMT remained apically localised despite the loss of MyoL signal (Supplementary Fig. 10a), indicating that TgMyoL is not required for TgPCKMT recruitment or stable positioning at the PCR-P2 region.
Together, our data support a model in which TgMyoL and TgPCKMT operate within the same structural framework at the PCR-P2 ring, but do not establish a strictly hierarchical relationship in which TgMyoL controls TgPCKMT localisation. Instead, these components likely act in a coordinated manner within a calcium-responsive regulatory hub at the conoid.

“Among the apical proteins detected in these datasets, TgMyoL was of particular interest because recent atomic models of the conoid PCR-P2 ring place TgPCKMT, referred to as AKMT2 in that study, in close proximity to TgMyoL and its associated calmodulin-like light chains4. This structural organisation suggests that TgPCKMT and TgMyoL occupy the same specialised region of the apical complex. Consistent with this, TgMyoL was detected in the TgPCKMT proximity-labelling dataset and was also enriched in the TgAKMT methylome dataset. We therefore examined whether TgMyoL contributes to TgPCKMT localisation. Upon TgMyoL depletion, TgPCKMT-Halo remained concentrated at the conoid despite loss of MyoL-YFP signal, indicating that TgMyoL is not required for TgPCKMT recruitment or stable positioning at the PCR-P2 region (Supplementary Fig. 10a).“ (Page 12, lines 263-272)

And

“Recent structural and functional studies provide a coherent framework for understanding how motility initiation is regulated at the apical complex. Cryo-electron tomography of the PCR-P2 ring has revealed a modular architecture

in which TgPCKMT, TgFRM1, and the myosin-like protein TgMyoL are positioned in close proximity, together with calmodulin and myosin light chain subunits, suggesting a localized platform for calcium-responsive regulation4,5. TgMyoL has been identified as a non-motor, calmodulin-associated component that mechanically tethers the preconoidal rings to the conoid and is important for conoid extrusion and gliding motility, while being dispensable for microneme secretion6. Together, these findings suggest that the TgMyoL–calmodulin complex forms a calcium-responsive structural element at the PCRs. However, TgPCKMT remains apically localised upon TgMyoL depletion, and published TgMyoL-depletion phenotypes are less severe than the near-complete block in motility initiation caused by TgPCKMT loss. These observations suggest that TgMyoL does not primarily regulate motility initiation through controlling TgPCKMT recruitment to the PCRs, and instead point to a coordinated, but not strictly hierarchical, relationship between these components within the PCR–conoid regulatory module."
(Page 18, lines 429-443)

(2) In PMID: 41366525, the authors report the localization of seven identified P2 essential proteins: FRM1, FLM1, FLM2, AKMT2 (PCKMT), PCR10, CAM4, and SEC24. This study also positions MyoL as part of the essential proteins in PCR-P2. The deletion of MyoL severely impaired parasite growth and invasion. Furthermore, the authors propose that the MyoL neck region interacts with three potential myosin light chain subunits (CAM1, CAM4, and MLC4) that contain calcium-binding EF-hand domains. Did the authors find these proteins in proximity labeling assays, and if so, would calcium signaling in this region be an additional step in the mechanism that leads to conoid extrusion? In this same context. Determining the role of calcium in motility initiation will strengthen the manuscript, given the critical role of calcium in this process, as demonstrated by the use of calcium ionophores. Furthermore, the elimination of MyoL may influence PCKMT activity during the initiation of motility.
We thank the reviewer for this insightful comment. Several PCR-P2 components are detected in our proximity-based datasets. TgMyoL is present in the TgPCKMT TurboID experiments and is also enriched in the TgAKMT methylome. In addition, the AKMT methylome shows enrichment of TgFLM1 and the calmodulin TgCAM4. In the PCKMT TurboID dataset, we detect most PCR-P2 components highlighted by the reviewer, with the exception of TgSEC24 and TgCAM4, suggesting that these factors may not be in close proximity to the C-terminally tagged PCKMT used for proximity labelling. Importantly, we note that proximity labelling does not imply direct physical interaction or substrate–enzyme relationships, as illustrated by the presence of additional proteins in the TurboID datasets that are unlikely to directly interact with TgPCKMT (Supplementary Fig. 10). We therefore interpret these data as reflecting spatial organisation within a densely packed apical complex rather than definitive evidence for direct molecular interactions or methylation events.
With respect to calcium signalling, we agree that calcium plays a central role in motility initiation in apicomplexan parasites as has been demonstrated several times over the years. Recent structural and functional studies position TgMyoL and associated calmodulin light chains within the PCR-P2 complex, raising the possibility that calcium-dependent structural changes occur at this site. However, direct calcium sensing by TgMyoL, or a mechanistic coupling between calcium signalling and TgPCKMT activity, has not yet been experimentally demonstrated.
Consistent with this, TgMyoL depletion does not alter TgPCKMT apical localisation, and the reported TgMyoL-depletion phenotypes are less severe than those observed upon loss of TgPCKMT. Together, these observations suggest that, while calcium-dependent regulation at the PCR-P2 complex is an important area for future investigation, defining how calcium signalling interfaces with TgPCKMT function lies beyond the scope of the present study.
(3) A major limitation is the identity of the directly methylated substrate. FRM1 is the most logical candidate. Methylation could block an autoinhibitory interaction or create a binding surface for the conoid scaffold. The report notes that actin itself was not enriched in methyl-peptide pulldowns, suggesting the regulation is at the level of the nucleator (FRM1) rather than the filament itself. Given the proximity, methylation of MyoL could stabilize the open conformation of the calcium sensor. Future experiments focusing specifically on the PCR-P2 complex components are required to identify the precise lysine residue(s) that act as the molecular switch.
We agree with the reviewer that identifying the direct methylation substrate(s) and the relevant lysine residue(s) represents an important next step. Based on our genetic, imaging, and proteomic data, TgFRM1 emerges as a functional effector of TgPCKMT in motility initiation. However, at present, we cannot distinguish whether TgPCKMT-dependent methylation primarily stabilises TgFRM1 positioning at the conoid, relieves autoinhibition, or contributes more directly to its activation state. Consistent with this

uncertainty, our data indicate that TgPCKMT-dependent methylation is required both for stable TgFRM1 anchoring and for productive actin nucleation, suggesting that methylation may serve a licensing function rather than acting solely as a binary on/off activation switch.

Actin itself was not enriched in our methyl-enrichment or TurboID datasets, which supports regulation at the level of the actin nucleator or associated PCR-P2 components rather than the bulk actin filament. However, we cannot exclude the possibility that actin undergoes low-stoichiometry or highly localised methylation that is difficult to detect against the large cellular pool of actin, particularly given reports of actin methylation in other organisms7-9. We therefore avoid ruling out actin methylation entirely and instead interpret our data as pointing primarily towards regulation of the nucleation machinery.

We therefore frame identification of the precise methylation site(s), direct substrate(s), and their temporal regulation during conoid assembly versus motility activation as important future directions. We have clarified this point in the Discussion to emphasise that defining the molecular methylation switch within the PCR-P2 complex lies beyond the scope of the present study:

"Strikingly, TgPCKMT operates with exceptional specificity: proximity labelling and methyl-enrichment identified only a limited number of candidate substrates, with TgFRM1 emerging as the most prominent effector linked to motility. Consistent with this focused role, the TgPCKMT-associated datasets were not broadly enriched for cytoskeletal motors or vesicle-organisation factors, distinguishing TgPCKMT from TgAKMT and supporting its function as a regulatory licensing factor rather than a motor-associated remodelling component. Loss of TgPCKMT disrupts TgFRM1 recruitment to mature conoids, and mutation of the catalytic residue H542 mirrors this phenotype, impairing conoid extrusion and gliding. These findings indicate that TgPCKMT's enzymatic activity, and to a certain extent its structural presence, are both required to license TgFRM1-dependent motility initiation. At present, whether distinct methylation events differentially regulate TgFRM1 positioning at the conoid versus its activation state remains unresolved. Defining the precise methylation site(s), direct substrate(s), and temporal regulation of these modifications during conoid assembly versus acute motility activation will therefore be an important future direction, but lies beyond the scope of the present study." (Page15, lines 353-366)

(4) The authors should integrate the following information in the results and discussion sections, where appropriate (perhaps the flow of information in the manuscript can be more coherent). PCKMT is stably associated with the PCRs. Its interactome (TurboID) is enriched for apical proteins, but surprisingly, it lacks many cytoskeletal motors, reflecting its specific role as a regulator rather than a motor component. AKMT is dynamically associated with the conoid. Its interactome is rich in myosin complex components, vesicle organization factors, and cytoskeletal proteins. This reflects its broader role in remodeling the pellicle during translocation. In PCKMT-depleted parasites, AKMT localization is aberrant. In resting, AKMT localizes to the conoid normally. Upon calcium stimulation, AKMT fails to release into the cytosol. This establishes the directionality of the cascade: PCKMT is upstream of AKMT. The release of AKMT appears not to be a direct response to calcium but is dependent on the actin polymerization licensed by PCKMT. Fluorescence Recovery After Photobleaching (FRAP) analysis highlights the dynamic nature of these proteins. GAC-YFP exhibits rapid recovery (12.8s), indicating a highly mobile pool that constantly exchanges with the cytosol. AKMT-YFP shows slower but significant recovery (42.3s). IMC1-Halo shows no recovery (structural control). The dynamic exchange of AKMT and GAC, even in the resting state, suggests the system is "primed." They are not cemented in place but are held in a dynamic equilibrium, ready to be biased towards translocation by the explosive polymerization of actin triggered by PCKMT.

We thank the reviewer for this helpful suggestion. We have now clarified the conceptual distinction between TgPCKMT and TgAKMT in both the Results and Discussion. Specifically, we emphasise that TgPCKMT is stably associated with the preconoidal rings and displays a restricted, apically enriched proximity/methylation profile consistent with a focused regulatory licensing role, rather than a broad motor-associated remodelling function. In contrast, TgAKMT is dynamically associated with the conoid and exhibits a broader methylation/proximity profile enriched in myosin complex components, cytoskeletal factors and vesicle organisation proteins, consistent with a downstream role in apical and pellicular remodelling.

We further clarify that TgAKMT localisation is normal in resting TgPCKMT-depleted parasites, but that TgAKMT fails to redistribute upon calcium stimulation. This establishes the directionality of the cascade, placing TgPCKMT upstream of TgAKMT mobilisation. Together with the TgFRM1-depletion and cytochalasin D experiments, these data indicate that TgAKMT release is not solely a direct response to calcium, but

depends on actin polymerisation licensed by the TgPCKMT–TgFRM1 axis. Finally, we explicitly integrate the FRAP analyses to show that TgAKMT and TgGAC dynamically exchange at the apical complex in resting parasites, while IMC1-Halo behaves as a stable structural control. These data support a model in which TgAKMT and TgGAC exist in a dynamic, primed equilibrium that can be biased toward productive translocation by TgPCKMT-dependent actin assembly.

(5) In wild-type parasites, apical actin fluorescence (CbHalo) is high, while in Cytochalasin D (CytD) treated parasites, it is low. The same assay for the catalytically dead (PCKMT-H542A) mutant can prove that the catalytic defect leads to a polymerization defect. Could the authors repeat the experiment using the same strain and determine the role of methyltransferase activity in actin polymerization and the initiation of motility?

We thank the reviewer for this suggestion. The CbHalo/Cytochalasin D experiment included in the manuscript was designed to test whether TgAKMT relocalisation is actin-dependent, rather than to directly measure the upstream licensing of actin polymerisation by TgPCKMT catalytic activity. We agree that monitoring CbHalo in the PCKMTH542A background could provide an additional visual readout of this pathway. However, the manuscript already provides evidence that the catalytic defect acts upstream of actin-dependent motility initiation: in the PCKMTH542A mutant, TgFRM1 is strongly reduced at mature conoids, closely resembling the TgPCKMT-depletion phenotype. Since TgFRM1 is the apical actin nucleator required for local F-actin formation during motility initiation, its failure to accumulate at the conoid provides a mechanistic basis for the expected impairment in local actin polymerisation in the catalytic mutant.

Consistent with this interpretation, the PCKMTH542A mutant fails to rescue conoid extrusion, egress and productive motility. Thus, although the suggested CbHalo experiment would provide an additional downstream readout, the existing TgFRM1 localisation, conoid extrusion, egress and motility data already support the conclusion that TgPCKMT catalytic activity is required upstream of actin-dependent motility initiation. We have therefore emphasised in the manuscript that TgPCKMT catalytic activity is required for stable TgFRM1 recruitment and for the downstream motility events that depend on local actin assembly.

Minor

(1) The manuscript should include the DiCre\Delta Ku80 parental control data in the invasion assay graph (Fig. 1i) to allow for direct normalization against the plaque and gliding assays.

We have revised Fig. 1i to include the DiCreΔku80 parental control alongside the TgPCKMT conditional knockout data. The invasion assay is now presented as a direct comparison between the parental and TgPCKMT-depleted parasites, allowing consistency with the plaque and gliding assays and facilitating interpretation of the invasion defect.

(2) Add a label to Figure 6a stating that it represents the condition with the ionophore so that it is not confused with Figure 4c, where the localization of AKMT is only observed in the conoid. The ambiguity in AKMT localization must be resolved by explicitly labeling panels as "+ Calcium Ionophore" versus "Resting."

We thank the reviewer for pointing out this ambiguity. We have now added “+CI” labels to Fig. 6a–d to indicate calcium ionophore-induced conditions and to clearly distinguish these panels from the resting localisation shown in Fig. 4c. We have also updated the Fig. 6 legend to state explicitly that these panels correspond to parasites stimulated with calcium ionophore.

(3) The quantification in Figure 6e should be refined to report "Cytosolic/Total Fluorescence Ratio" rather than a binary "Translocated" score, to better capture the nuance of partial defects in mutants.

We thank the reviewer for this helpful suggestion. We have now added a conoid/cytosolic AKMT fluorescence ratio quantification in Fig.6f, which better captures the relative enrichment of AKMT at the conoid and allows partial redistribution defects in the mutant lines to be assessed more quantitatively.

We have also retained the quantification of the percentage of parasites in which AKMT is no longer detected at the conoid in Fig.6e, as this remains an informative complementary measure of AKMT translocation.

(4) Strict adherence to strain labels (e.g., loxPpckmt-Halo vs. DiCre) in figure legends is essential to avoid confusion between wild-type and non-induced controls. Please include the plaque number quantification in the supplementary material. Figure 2 b and c: Please label the strains used.

We have revised the figure legends and labels to ensure strict and consistent use of strain names, including distinguishing the DiCreΔku80 parental control from non-induced loxPpckmt-Halo parasites. We have also added the strain labels to Fig. 2b,c. Regarding plaque quantification, we did not include numerical plaque counts because TgPCKMT-depleted parasites reproducibly failed to form detectable plaques under the

assay conditions. Thus, the phenotype was essentially binary and plaque number quantification would not provide an additional meaningful measure beyond the representative plaque assay shown. We have clarified this point in the figure legend/text to indicate that no plaques were detectable upon TgPCKMT depletion.
(5) To maintain consistency in the figures, the authors could place the boxes indicating the magnification in all the figures; for example, they are indicated in Figure 1d, but not in Figure 1e.
We have now added magnification boxes consistently across the relevant figures, including Fig. 1e, Fig. 3e and Fig. 6a–d, to improve clarity and maintain a uniform figure style.
(6) Line 304, Figure 6b should be in the place of Figure 6a.
Corrected
(7) Figure 6e is missing the y-axis label.
We have added a label to the y-axis in Fig 6e
(8) Line 309, Figure 6b, should be Figure 6c. Please change accordingly.
Corrected
(9) Line 312, Figure 6c should be 6d.
Response to (6)(8)(9): We apologise for this mistake. The figure numbering and corresponding callouts have now been corrected consistently throughout the manuscript.
(10) Lines 319-322: The quantification they performed is not clear. The images in Figures 6a and 6 b show AKMT in both the apical region and the cytosol; however, this is not reflected in the quantification in Figure 6e.
As noted above, we have now revised the quantification to include the relative TgAKMT intensity ratio between the conoid/apical region and the parasite body/cytosol. This better reflects the redistribution of TgAKMT observed in the images and distinguishes parasites with predominantly apical TgAKMT from those showing increased cytosolic signal. We have also updated the Results text and figure legend to explicitly describe this ratio-based quantification.
"We quantified these dynamics using two complementary approaches. First, we scored the percentage of vacuoles showing TgAKMT translocation across all conditions, including only parasites deficient for the floxed genes analysed above or treated with CytD to abolish actin polymerisation (Fig. 6e). Second, to capture the mixed apical and cytosolic TgAKMT signal observed in some conditions, we measured the relative TgAKMT intensity at the conoid versus the parasite body/cytosol (Fig. 6f). This ratio-based analysis confirmed that TgPCKMT- and TgFRM1-depleted parasites, as well as CytD-treated parasites, retain high conoid-to-cytosol TgAKMT ratios, consistent with impaired mobilisation. By contrast, TgGAC-depleted parasites showed reduced ratios, consistent with TgAKMT disengagement from the conoid despite altered cytosolic distribution. Together, these results indicate that TgAKMT redistribution depends on the TgPCKMT–TgFRM1–actin axis but occurs independently of TgGAC." (page 13-14, lines 315-325)
Reviewer #3:
Summary
This study reports on a novel methyl transferase (PCKM1) identified in T gondii, with a similar function in P. falciparum. Localization of PCKM1 at the pre-conoidal ring is largely independent from other know PCR proteins, whereas only FRM1 localization to the conoid appears to be dependent on PCKM1 activity, and there is no effect on the stability of the cytoskeleton. Hence, a very stringent 1 on 1 relationship, and it is remarkable that this so nicely came together here (wide methylome and TurboID nets were cast). I appreciated the detail that FRM1 is normally recruited to the PCR during budding, but that PCKM1 causes a maturation to retain FRM1 in mature parasites. Mechanistically, the model presented in Fig 7 says is all: PCKM1 recruits FRM1 to promote conoid extrusion during parasite egress (downstream of Ca and cGMP kinases), which in turn is required to start actin polymerization needed for gliding motility through GAC and AKMT translocation from the conoid to cytoplasm. Although new questions arise as nicely laid out in the discussion, this study is a major mechanistic leap for the field. Overall, this is a very well-designed study including all controls and quantitative assessments where possible. I am on board with result interpretations and assembled model. However, I have many relatively minor remarks on how data are presented or are collected
We thank Reviewer #3 for this thoughtful and encouraging assessment of our work. Below, we address the reviewer's specific comments regarding data presentation and clarity.
Specific points
1. Please provide the ToxoDB GeneID in the main manuscript (finally found in Table S3).
We have added the gene ID in the first result section now (Page 4 line 67).

2. Many legends do not explain the size of the scalebar shown. Please add throughout all panels/legends.
We have revised the figure legends and added scale bar sizes wherever they were missing.
3. Fig S2a. An important detail is not explained very well in the manuscript. Looks like these parasites are undergoing budding, as 2 ICAP16 and PCKMT spots in each parasite are seen that are not in conoid, which I assume are daughters. Unfortunately, TLAP1 does not report on the presence of daughter buds. Digging in the literature, I could not find any details on ICAP16 in assembling daughters/conoids. Hence, some additional explanation or exploration in daughter assembly would be very welcome here (e.g. using an early daughter bud reporter like ISP1, or centrosome marker which duplicates before budding starts). Reading further, Fig 3f covers this aspect somewhat, but not in the ICAP16 context...
We thank the reviewer for this thoughtful observation. The additional ICAP16 and PCKMT puncta visible in Fig. S2a indeed correspond to assembling daughter cells. As shown previously (Li et al., 2025)3, preconoidal ring components are recruited very early during daughter cell formation, prior to completion of conoid assembly. Although ICAP16 was not specifically analysed in that study, or in the present one, its behaviour in Fig. S2a is consistent with the early recruitment dynamics reported for other preconoidal ring proteins. To make this clearer, we have now added arrows to Fig. S2a to distinguish mature conoids from developing daughter conoids and have clarified this in the corresponding figure legend.
The purpose of Fig. S2a in the present manuscript is to illustrate the localisation of PCKMT and ICAP16 to preconoidal ring structures in both mature and developing parasites, rather than to provide a detailed analysis of their dynamics during daughter cell assembly. A systematic investigation of ICAP16 recruitment using early daughter bud markers or centrosomal markers would be required to address this question comprehensively, but we consider this beyond the scope of the current study and outside the central focus on PCKMT function in motility initiation.
4. Fig S2b-e. It would be helpful to add the parasite strain in the figure itself (now it is in the legend). Video 3 is properly labeled, so apply the same here.
We have inserted labels in these panels now.
5. Fig 1h. These plaque assays are not the clearest image as it is hard to make out plaques, and the difference with +Rapa LoxPpckmt is very subtle (e.g. I see 2 clearly white spots of different size in Dicre -Rapa, but I do not think those are plaques?). Please replace with better stained plaque assay. Fig 3a is marginally better, but could also benefit from clearer panels
We have replaced the representative plaque assay images in Fig. 1h with clearer, better stained panels in which plaques are more readily visible. We have also improved the presentation of the complementation plaque assay panels, now shown in Supplementary Fig. 6a.
6. Fig 2b-d. Please add the color-key for factors visualized in the figure panels rather than the legend. Panel 2d legend does not explain what blue and magenta represent at all (the blue looks like the moving junction???). Colors are also not explained for Fig S6b and c.
We have now added explicit colour keys directly to the relevant figure panels for Fig. 2b–d and Supplementary Fig. S6b,c, as suggested. In all cases, the blue signal corresponds to parasite nuclei. The unusual morphology observed in some panels likely reflects the expansion process and the distribution of DNA within the expanded nuclei. These clarifications have been added to the figures and legends to improve clarity.
7. Line 164-166. This sentence would be better as an opener in the next section. A stylistic opinion?
We agree and have moved this sentence to the beginning of the following section, as suggested.
8. Fig S5a. I am trying to understand what exactly was done here. the number of the PCRs, do they represent the order? “5. Insertion PCR” only has 1, slanted arrow, which I am not sure at all how to interpret? I thought this construct was targeted into the UPRT locus, but the pGEM-T flanks seem to suggest otherwise?? Or is this a Plasmodium schematic?
We apologise that Fig. S5a and its legend were not sufficiently clear. We have revised both the schematic and the corresponding legend to clarify that this figure shows the generation and validation of the TgPCKMT complementation and catalytic-mutant parasite lines in T. gondii, not Plasmodium. We now specify that the numbered labels refer to the cloning and validation steps shown in the subsequent gel panels, rather than to a strict chronological order of PCR reactions.
We have also clarified that the pGEM-T sequences shown in the schematic represent the cloning backbone, whereas the complementation constructs were targeted to the T. gondii UPRT locus. Finally, we have revised the label for the insertion PCR to make

clear that it corresponds to diagnostic PCR validation of parasite integration.
9. Fig S6b,c. Please explain in the legend what the white arrows point at.
We have added an explanation in the figure legend.
10. Fig S8c,d legends. These western blots do not permit the conclusion that in c apical and cytosolic proteins are biotinylated, and in d preconoidal and apical apicomplex-associated proteins. Please revise legends (e.g. remove these comments, or refer to Fig 5 corresponding BioID panels).
We have revised the Supplementary Fig. 8 legend to avoid assigning the streptavidin-detected bands to specific subcellular protein classes. The legend now describes panels b–d as validation of TurboID-dependent biotinylation, while interpretation of the corresponding protein categories is restricted to the proteomics data shown in Fig. 5.
11. Fig 5d, f, h. Please add in the panel whether methylome or biotinylation is plotted.
We have now added labels to Fig. 5d, f, and h specifying whether each panel represents methylation or proximity labeling data.
12. Fig 10b. TGGT1_212780 was described as SFA5 (https://doi.org/10.1186/s44280-024-00043-1); ASAF1 was assigned SFA4 in the same paper. Looks like SFA5 might indeed be an ASF component, but that ASAF1 is not confirmed on the SFA?
We thank the reviewer for raising this important nomenclature point. We have corrected the labelling in Supplementary Fig. 10b and the associated legend to ensure consistency, and we have now added references in the main text to both the published study cited by the reviewer and the recent bioRxiv study from the Lebrun laboratory.
In the recent study by Li et al., the protein we refer to here as ASAF1 was defined and characterised as a critical factor for early apical polar ring (APR) and nascent conoid complex assembly. This protein corresponds to TGGT1_212780, which has also been referred to as SFA5 in the study cited by the reviewer. That work demonstrated that ASAF1/SFA5 is important for APR assembly and its functional linkage to centrosomes via SFA2.
In the context of the present manuscript, Fig. 10 is included solely to illustrate the localisation of selected proteins identified in the TgPCKMT proximity-labelling datasets, highlighting their association with the conoid or developing apical structures. We do not further analyse the functional role of ASAF1/SFA5 in this study.
13. Fig 6e has no y-axis label. It is not immediately clear how AKMT translocation was quantified – signal intensity or an arbitrary score? The source data suggests and arbitrary score but the description there is not clear either. Please clarify
We have added a y-axis label to Fig. 6e and clarified in the legend and source data that AKMT translocation was quantified as the percentage of parasites lacking detectable AKMT at the conoid.
14. Line 329 refers to Fig 6d,e. which should be 6e,f. Movie S7 was not playing for me (all others did), but Fig e,f are very clear.
We apologise for the incorrect figure reference in the text, which has now been corrected to Fig. 6e,f. We have also re-uploaded Movie S7 and hope that this has resolved the playback issue.

Reviewer #4
I co-reviewed this manuscript with one of the reviewers who provided the listed reports. This is part of the Nature Communications initiative to facilitate training in peer review and to provide appropriate recognition for Early Career Researchers who co-review manuscripts.
We thank Reviewer #4 for their contribution to the review of this manuscript. We greatly appreciate this Nature Communications initiative and find it an excellent and forward-looking approach to supporting and recognizing Early Career Researchers in peer review. We hope that many Early Career Researchers will benefit from this initiative.

1 Brancucci, N. M. B. et al. Probing Plasmodium falciparum sexual commitment at the single-cell level. Wellcome Open Res 3, 70 (2018). https://doi.org:10.12688/wellcomeopenres.14645.4
2 Bertiaux, E. et al. Expansion microscopy provides new insights into the cytoskeleton of malaria parasites including the conservation of a conoid. PLoS biology 19, e3001020 (2021). https://doi.org:10.1371/journal.pbio.3001401
3 Li, W. et al. An apical ring protein essential for conoid complex assembly and daughter cell formation in Toxoplasma gondii. Nature Communications 16, 10149 (2025). https://doi.org:10.1038/s41467-025-65382-y
4 Zeng, J. et al. Atomic models of the Toxoplasma cell invasion machinery. Nat Struct Mol Biol 33, 157-170 (2026). https://doi.org:10.1038/s41594-025-01728-w
5 Qian, P. et al. Evolutionarily convergent mechanism of formin regulation coordinates actin polymerization in apicomplexan parasites. Science advances 11, eaea2136 (2025). https://doi.org:10.1126/sciadv.aea2136
6 Haase, R. et al. MyoL-Dependent Coupling of Preconoidal Rings to Conoid Is Required for

Motility in *Toxoplasma gondii*. bioRxiv, 2026.2001.2006.697858 (2026).
https://doi.org:10.64898/2026.01.06.697858
7 Kumar, A. et al. Actin R256 Mono-methylation Is a Conserved Post-translational Modification Involved in Transcription. Cell reports 32, 108172 (2020).
https://doi.org:https://doi.org/10.1016/j.celrep.2020.108172
8 Kwiatkowski, S. et al. SETD3 protein is the actin-specific histidine N-methyltransferase. Elife 7 (2018). https://doi.org:10.7554/eLife.37921
9 Seervai, R. N. H. et al. The Huntingtin-interacting protein SETD2/HYPB is an actin lysine methyltransferase. Sci Adv 6 (2020). https://doi.org:10.1126/sciadv.abb7854

Version 1:

Reviewer comments:

Reviewer #1

(Remarks to the Author)
I thank the authors for their thoughtful response, and I have no further comments.
Congratulations on a beautiful study.

Reviewer #2

(Remarks to the Author)
The authors have addressed my concerns. The work has significantly improved and worthy of a publication.

Reviewer #3

(Remarks to the Author)
The authors have been very responsive to the review and addressed all my previous concerns effectively.

Open Access This Peer Review File is licensed under a Creative Commons Attribution 4.0 International License, which permits use, sharing, adaptation, distribution and reproduction in any medium or format, as long as you give appropriate credit to the original author(s) and the source, provide a link to the Creative Commons license, and indicate if changes were made.
In cases where reviewers are anonymous, credit should be given to 'Anonymous Referee' and the source.
The images or other third party material in this Peer Review File are included in the article’s Creative Commons license, unless indicated otherwise in a credit line to the material. If material is not included in the article’s Creative Commons license and your intended use is not permitted by statutory regulation or exceeds the permitted use, you will need to obtain permission directly from the copyright holder.
To view a copy of this license, visit https://creativecommons.org/licenses/by/4.0/
