# Supplementary material for: Dual apical methyltransferases orchestrate motility initiation in apicomplexan parasites
Source: Nat Commun. 2026 Aug 26;17:9068. doi: 10.1038/s41467-026-77078-y (PMC13518913; doi:10.1038/s41467-026-77078-y)

Supplementary Figures

Dual apical methyltransferases orchestrate motility initiation in apicomplexan parasites

Peipei Qin[1], Thrishla Kumar[1], Oliwia Koczy[2,3], Wei Li[1,4], Ignasi Forne[5], Simone Mattei[2,6], Elena Jimenez-Ruiz[1]

[1] Experimental parasitology. Faculty of Veterinary Medicine. Ludwig-Maximillian-University (LMU). Munich Germany

[2] European Molecular Biology Laboratory (EMBL), Molecular Systems Biology Unit, Heidelberg, Germany

[3] Collaboration for joint PhD degree between EMBL and Heidelberg University, Faculty of Biosciences

[4] Department of Parasitology, College of Veterinary Medicine, Sichuan Agricultural University, Chengdu, China

[5] Protein Analysis Unit, Faculty of Medicine, Biomedical Center (BMC), Ludwig-Maximilians-University (LMU), Munich, Germany

[6] European Molecular Biology Laboratory, EMBL Imaging Centre, Heidelberg, Germany

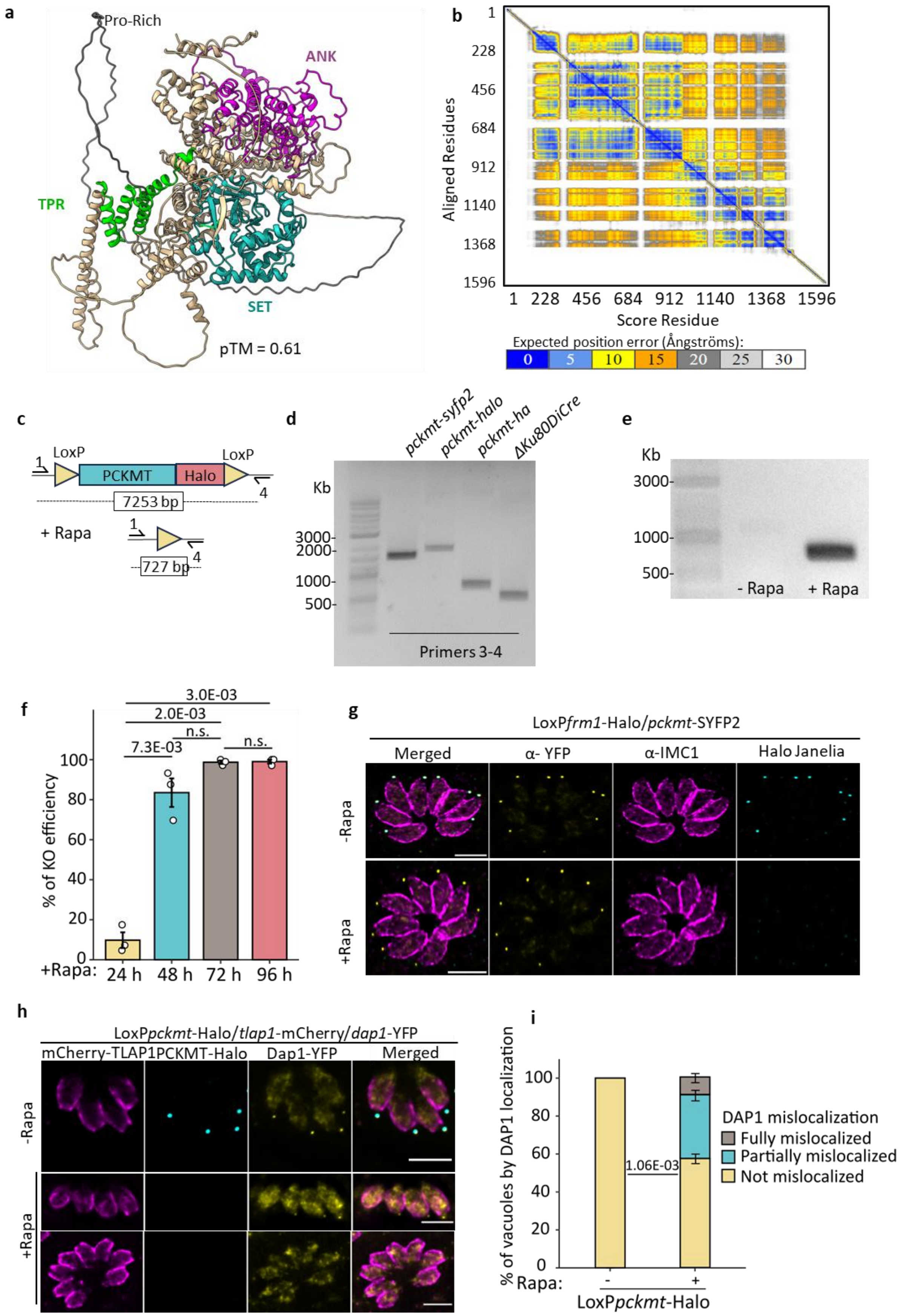

Supplementary Fig. 1 | Structure prediction and genetic analysis implicate PCKMT in conoid organisation and selective anchoring of FRM1 and DAP1.

(a) *AlphaFold2*-predicted structure of PCKMT showing the ordered domains, TPR repeats (green), SET catalytic core (cyan), and ankyrin repeats (magenta), connected by extended linkers predicted with low

confidence (beige). The predicted template modelling score (pTM = 0.61) indicates that while the overall domain topology is likely correct, the relative domain orientation may be uncertain.
(b) Predicted aligned error (PAE) heat map from *AlphaFold3* (accessed via https://alphafoldserver.com) for full-length PCKMT. The model reveals four well-defined regions: TPR, ankyrin, SET, and a proline-rich C-terminal segment. Colours indicate expected positional error between residue pairs (0–30 Å; blue = high confidence, yellow = low). The continuous low-PAE diagonal and distinct blue blocks correspond to intra-domain regions of high structural confidence, whereas warmer inter-domain regions denote flexible or poorly defined relative orientations.
(c–e) Schematic representation of the engineered *pckmt* locus (c), analytical PCRs confirming successful insertion of SYFP2, Halo, and HA tags (d), and excision of *pckmt* following 50 nM rapamycin treatment (e). The image shown is representative of three independent experiments.
(f) Quantification of knockout (KO) efficiency at different time points after induction with rapamycin. Bars represent mean ± standard deviation (SD), with dots indicating the mean of each biological replicate (n = 3).
(g) Immunofluorescence analysis of PCKMT localisation in *loxPfrm1* parasites shows that PCKMT localises to the conoid independently of FRM1. Scale bars, 5 µm. The image shown is representative of three independent experiments.
(h, i) Immunofluorescence analysis of DAP1 localisation in *loxPpckmt* parasites shows partial DAP1 mislocalisation upon PCKMT depletion (h). Scale bars, 5 µm. The image shown is representative of three independent experiments. (i) Quantification of DAP1 mislocalisation after 72 h of rapamycin treatment (50 nM). Data represent in stacked bar graph as mean ± SD from three biological replicates (n = 3). Source data are provided as a Source Data file.
Statistical significance in all quantifications was assessed by a two-tailed Student's *t*-test (n.s., not significant).
Source data are provided as a Source Data file.

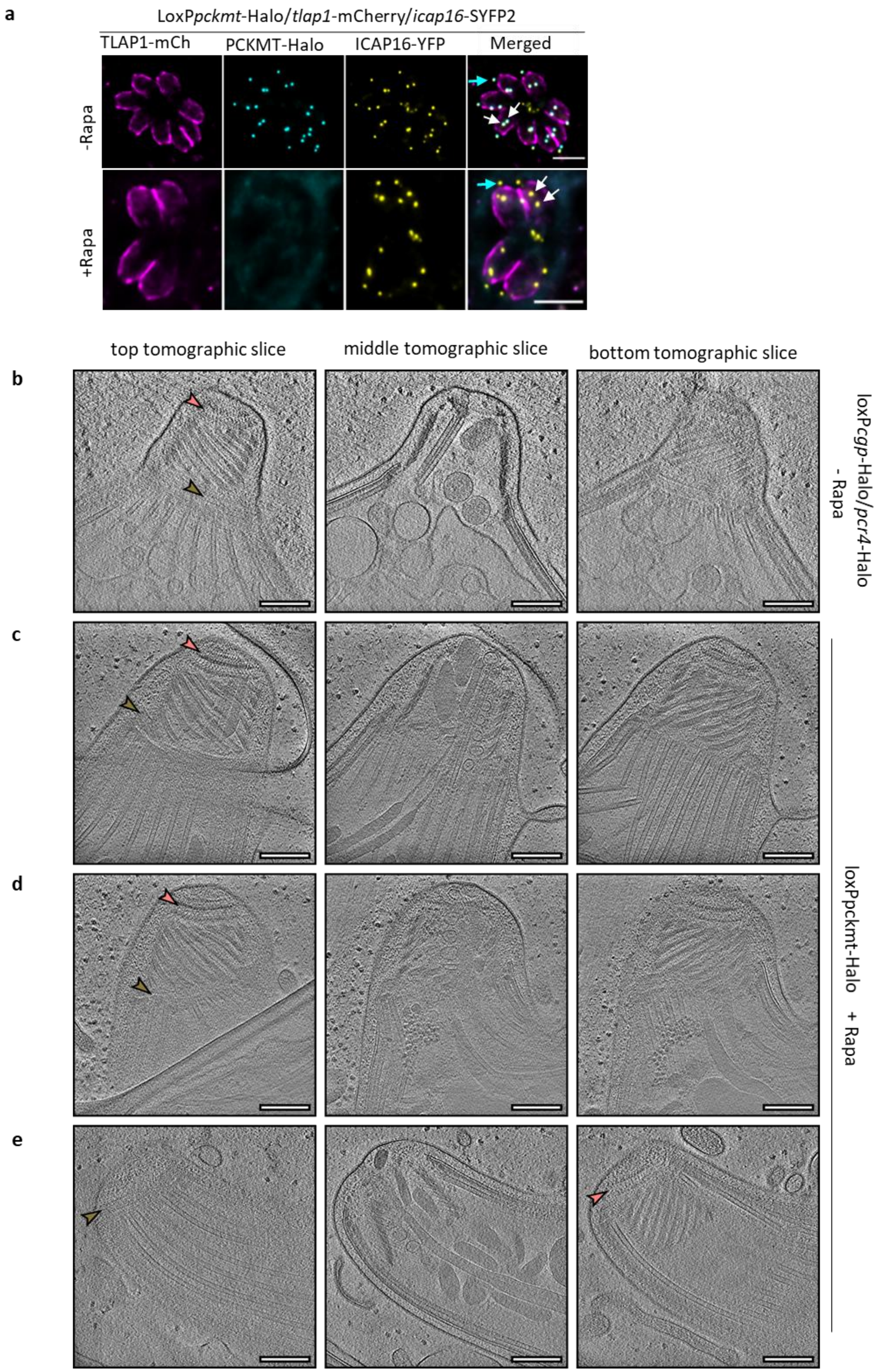

Supplementary Fig. 2 | ICAP16 localises independently of PCKMT, and cryo-ET shows preserved preconoidal ring/APR architecture in extruded *T. gondii* PCKMT iKO parasites.

(a) Immunofluorescence analysis of ICAP16 localisation in loxP*pckmt* parasites shows ICAP16 is independent of PCKMT. Scale bars, 5 µm. Blue arrows: mature conoids; white arrows: developing daughter conoids. The image shown is representative of three independent experiments.
(b-e) Reconstructed tomograms of *T.gondii* parasites presented as three example slices coming from the top, middle, and bottom of the reconstructed volume. Pink arrows indicate the PCRs, while khaki arrows indicate the APR. Scale bar: 200 nm. For full tomographic reconstructions, see Supplementary Video 3. **(b)** Tomogram of extruded WT parasite (loxP*cgp*-Halo/*pcr4*-Halo strain). **(c)** and **(d)** Tomograms of extruded iKO parasites (loxP*pckmt*-Halo strain). **(e)** Tomogram of non-extruded PCKMT iKO parasites (loxP*pckmt*-Halo strain).
The images shown are representative of the tomograms suitable for analysis (PCKMT iKO, n = 60 of 89 acquired tomograms; WT, n = 25 of 46 acquired tomograms).

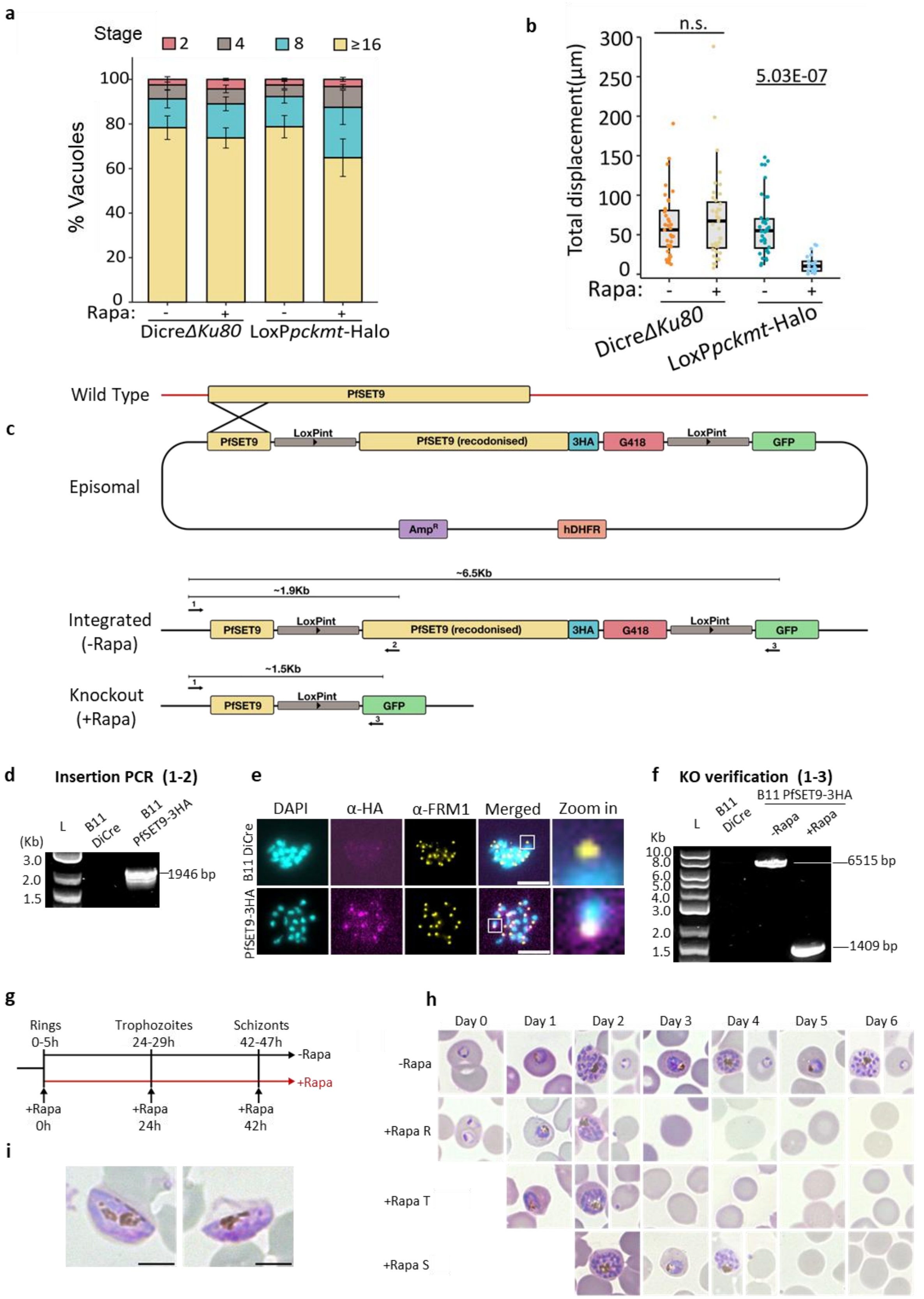

Supplementary Fig. 3 | Functional assays and genetic engineering show stable replication but impaired gliding after PCKMT depletion and validate the PfSET9 inducible knockout.
(a) Replication assay of *T. gondii* PCKMT-iKO parasites pre-induced with or without 50 nM rapamycin for 72 h and grown on HFF monolayers for 24 h. Vacuoles containing 2, 4, 8 or 16 parasites were counted; at least 100 vacuoles were analysed per replicate across three independent biological replicates. Data are presented as mean ± SD. Source data are provided as a Source Data file.

(b) Gliding-distance analysis of parasites pre-induced with or without 50 nM rapamycin for 72 h. Tracks were extracted from time-lapse recordings and measured in ImageJ. At least nine parasites were analysed per replicate across three independent biological replicates. Dots represent individual parasites; box plots show the median, interquartile range and 1.5× interquartile-range whiskers. Statistical significance was assessed using an unpaired, two-tailed Student's *t*-test (n.s., not significant). Source data are provided as a Source Data file.
(c) Schematic of the PfSET9-3HA inducible knockout strategy in *P. falciparum*, showing the wild-type locus, integration construct, modified locus and rapamycin-induced excision. Primers 1–3 were used for integration and knockout verification.
(d) PCR verification of construct integration using primers 1 and 2. B11 DiCre denotes the parental 3D7 line expressing DiCre.
(e) Immunofluorescence analysis of segmented schizonts showing apical PfSET9-3HA colocalisation with PfFRM1. Scale bar, 5 µm.
(f) PCR verification of PfSET9 excision 48 h after induction with 100 nM rapamycin.
(g) Experimental scheme showing induction of tightly synchronised parasites at ring, trophozoite or schizont stages. Non-induced cultures served as controls.
(h) Representative Giemsa-stained smears of cultures induced at ring, trophozoite or schizont stages, showing severe invasion defects after PfSET9 depletion.
(i) Representative early gametocyte-like parasites observed in induced cultures. Scale bar, 2 µm.

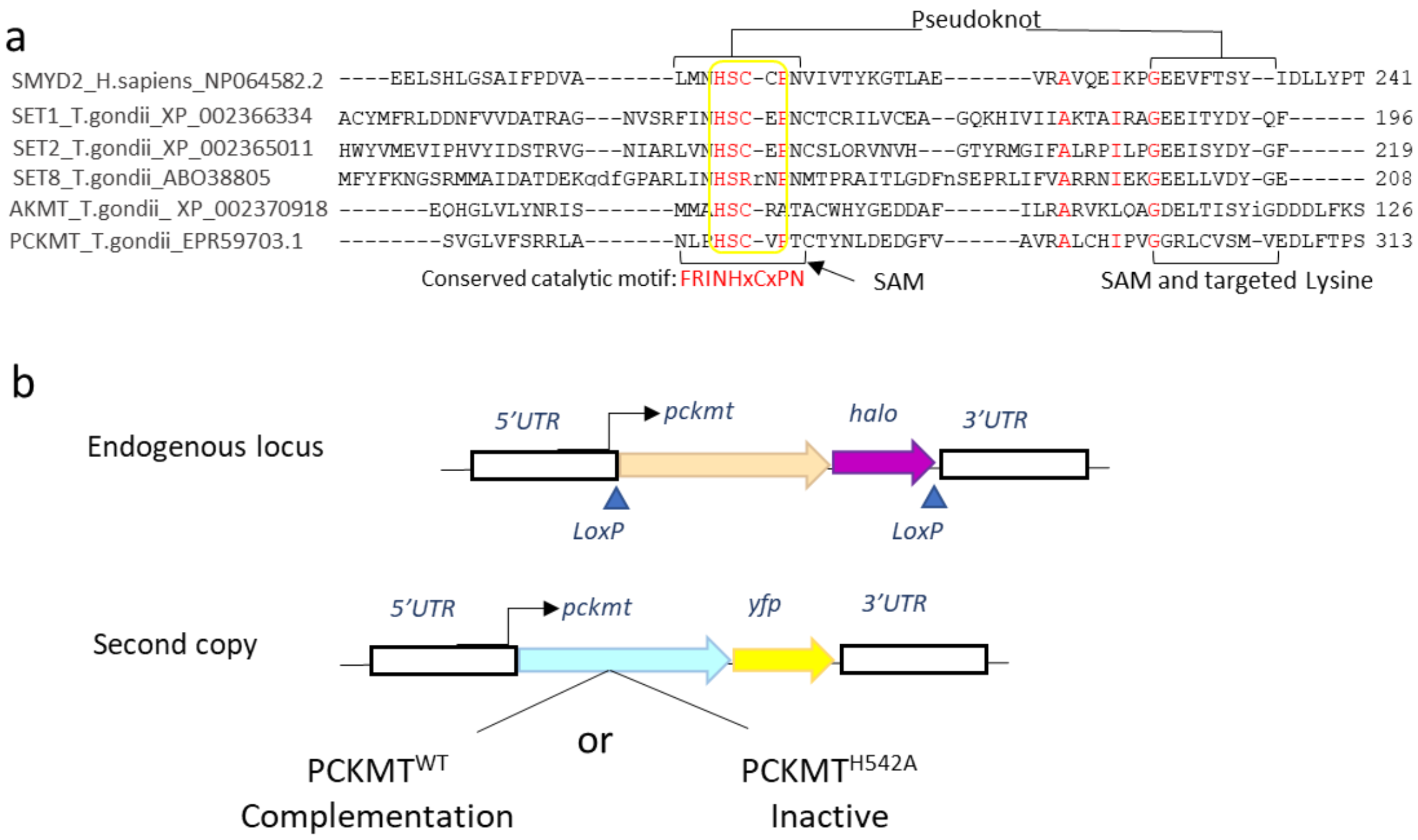

Supplementary Fig. 4 | Conservation of the PCKMT catalytic SET domain and generation of a complementation system for functional analysis.

(a) Multiple sequence alignment of the SET domain from *T. gondii* methyltransferases showing conservation of the catalytic motif HxC in PCKMT.

(b) Schematic of the complementation construct in which *pckmt–syfp2* was integrated into the *UPRT* locus under the control of the endogenous *PCKMT* promoter in the inducible *loxPpckmt–Halo* strain.

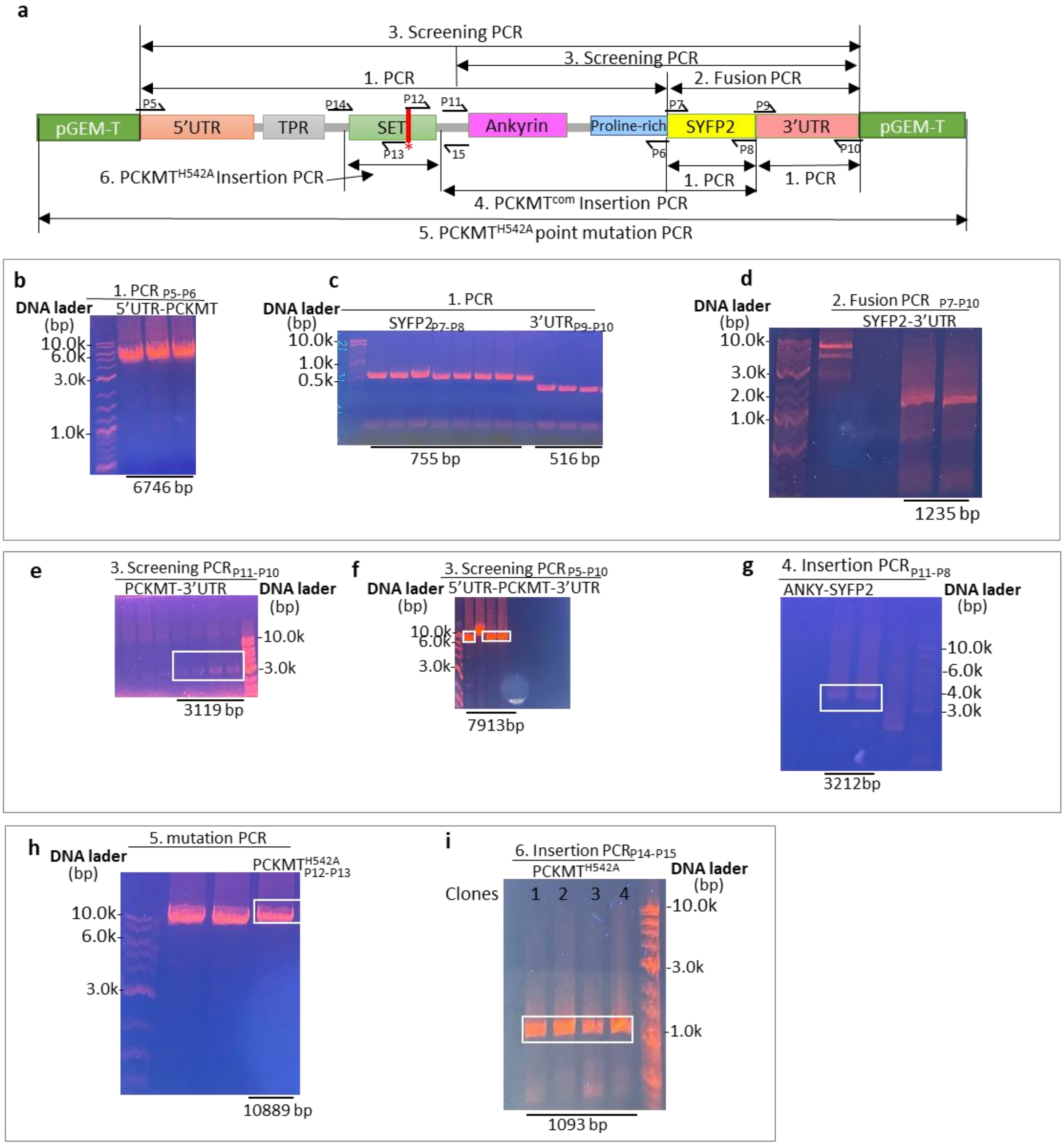

Supplementary Fig. 5 | Construction and validation of TgPCKMT-SYFP2 complementation and catalytic-mutant vectors.

(a) Schematic representation of the pGEM-T–TgPCKMT-SYFP2 complementation construct used to generate the TgPCKMT^{com} line and the TgPCKMT^{H542A} catalytic mutant. The construct contains the native TgPCKMT 5′ UTR, the TgPCKMT coding sequence lacking the endogenous stop codon, an in-frame C-terminal SYFP2 tag, and the TgPCKMT 3′ UTR cloned into the pGEM-T backbone. The position of the H542A catalytic mutation within the SET domain is indicated in red.

(b–d) Assembly of the TgPCKMT-SYFP2 complementation construct. PCR amplification was used to generate the individual fragments required for cloning, including the 5′ UTR–TgPCKMT fragment, the SYFP2 coding sequence, and the TgPCKMT 3′ UTR. The SYFP2 and 3′ UTR fragments were subsequently joined by fusion PCR to generate the SYFP2–3′ UTR fragment used for Gibson assembly with the 5′ UTR–TgPCKMT fragment into the pGEM-T backbone. Expected PCR products are indicated by boxes.

(e–f) PCR screening of bacterial colonies and analytical PCR validation of the assembled pGEM-T–TgPCKMT-SYFP2 plasmid. Positive clones were identified by PCR across the cloned junctions and by amplification of the expected full-length TgPCKMT-SYFP2 region. Boxed bands indicate clones with the expected amplicon sizes selected for downstream validation.

(g) Diagnostic PCR confirming integration of the TgPCKMT^{com}-SYFP2 complementation construct into the loxP*pckmt*-Halo/*frm1*-3HA parasite line. The expected integration-specific PCR product is boxed.
(h) Generation and validation of the pGEM-T–TgPCKMT^{H542A}-SYFP2 catalytic-mutant construct. The H542A mutation was introduced by point-mutation PCR using the pGEM-T–TgPCKMT-SYFP2 complementation plasmid as template. Candidate mutant plasmids were screened by PCR, and positive constructs were confirmed by Sanger sequencing prior to parasite transfection. Boxed bands indicate PCR products of the expected size.
(i) Diagnostic PCR confirming integration of the TgPCKMT^{H542A}-SYFP2 second-copy construct into the UPRT locus of the loxP*pckmt*-Halo/*frm1*-3HA parasite line. The expected integration-specific PCR product is boxed.

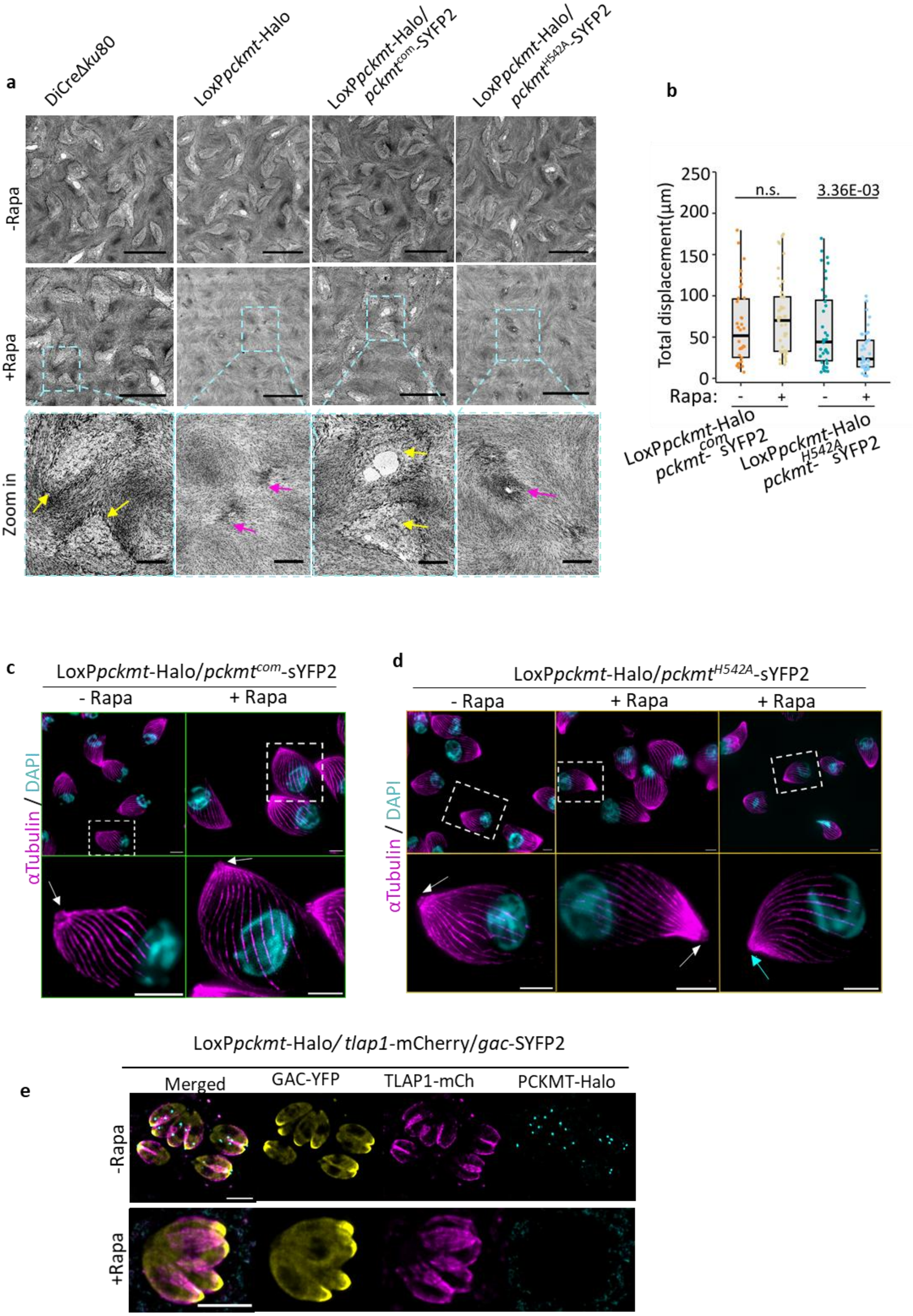

Supplementary Fig. 6 | Catalytic activity of PCKMT is required for anchoring FRM1 and initiating parasite motility.

(a) Representative plaque assays of *T. gondii* DiCreΔ*Ku80* (parental), loxP*pckmt*-Halo, loxP*pckmt*-Halo/PCKMT^{com} (complemented), and loxP*pckmt*-Halo/PCKMT^{H542A} (catalytic-dead) strains after ~7 days ± 50 nM rapamycin (Rapa). Scale bars in the upper and middle panels, 5 mm; scale bars in the

bottom panels, 500 µm. Yellow arrows indicate normal plaque formation. Magenta arrows indicate the defective plaque formation once PCKMT is depleted.
(b) Quantification of gliding motility in *T. gondii* tachyzoites expressing wild-type PCKMT or the catalytic mutant $PCKMT^{H542A}$. Gliding distances were measured from time-lapse recordings using ImageJ. Each point represents an individual parasite (at least 9 per replicate, n = 3 biological replicates); Box plots show the median as the centre line and the 25th and 75th percentiles as the lower and upper bounds of the box. Whiskers extend to the smallest and largest values within 1.5 times the interquartile range, and points beyond the whiskers represent outliers. Dots represent individual parasites. Statistical analysis was performed using an unpaired, two-tailed Student's *t*-test. n.s., not significant
(c-d) Representative ExM images of conoid extrusion. (c) in complemented parasites ($PCKMT^{com}$). Rapamycin-induced excision of endogenous *pckmt* caused a conoid extrusion defect, which was rescued by the expression of $PCKMT^{com}$ **(d)** in the catalytic mutant $PCKMT^{H542A}$. The point mutation failed to restore conoid protrusion following *pckmt* excision. Regions indicated by white boxes are enlarged and shown in the lower panels. White arrows indicate the extruded conoid, cyan arrow indicates the non-extruded conoid. Scale bar, 5 µm.
(e) Endogenous tagging of GAC in the conditional loxP*pckmt* strain shows that GAC remains unaffected by PCKMT loss in intracellular parasites, indicating a unidirectional dependency. Scale bar, 5 µm.
Source data are provided as a Source Data file. The images shown are representative of three independent experiments.

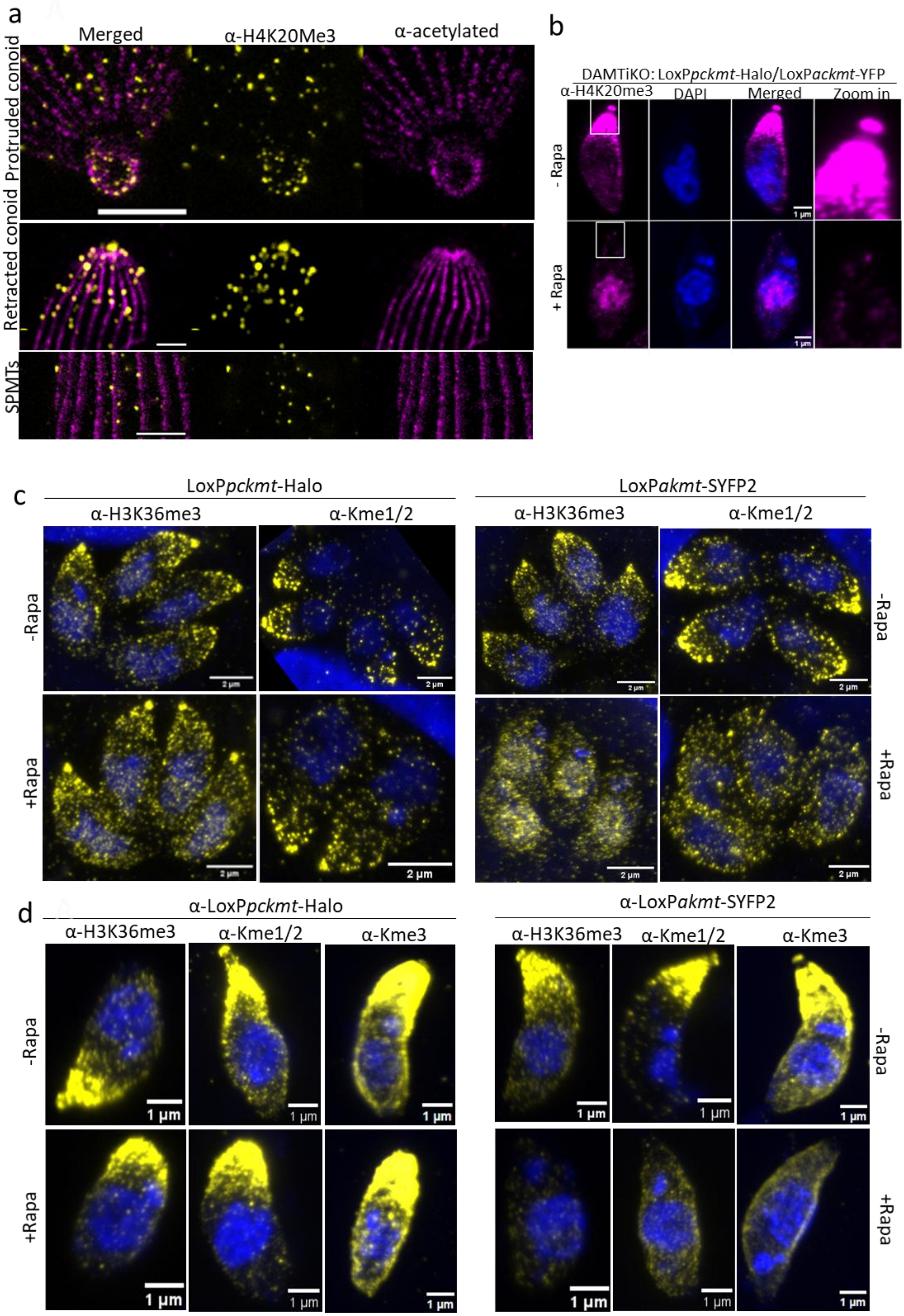

Supplementary Fig. 7 | Apical methylation resolved by U-ExM/STED reveals AKMT as the dominant lysine methyltransferase, with minimal contribution from PCKMT.

(a) U-ExM combined with STED further enhances the resolution of the methylation signal, preserving distinct labelling at the preconoidal rings and along microtubules after expansion. Scale bar, 2 µm.

(b) Immunofluorescence analysis of methylation using anti-H4K20me3 antibody in parasites induced with and without rapamycin (Rapa). Double knockout (DAMTiKO; AKMT/PCKMT). Loss of AKMT abolished the H4K20me3 signal at the apical complex, and the double knockout mirrored this phenotype, confirming AKMT as the dominant methyltransferase responsible for this modification. Scale bar, 1 µm.

(c) Intracellular tachyzoites immunolabelled with anti-H3K36me3 and anti-lysine mono/di-methyl (Kme1/2) antibodies. Bulk methylation levels decrease markedly upon AKMT depletion, whereas PCKMT depletion shows no detectable change. Scale bar, 2 µm.

(d) Extracellular tachyzoites stained with anti-H3K36me3, anti-Kme1/2, and pan-methyl-lysine antibodies display similar patterns, confirming AKMT as the dominant methyltransferase responsible for global methylation signals**.** Scale bar, 1 µm.

The images shown are representative of three independent experiments.

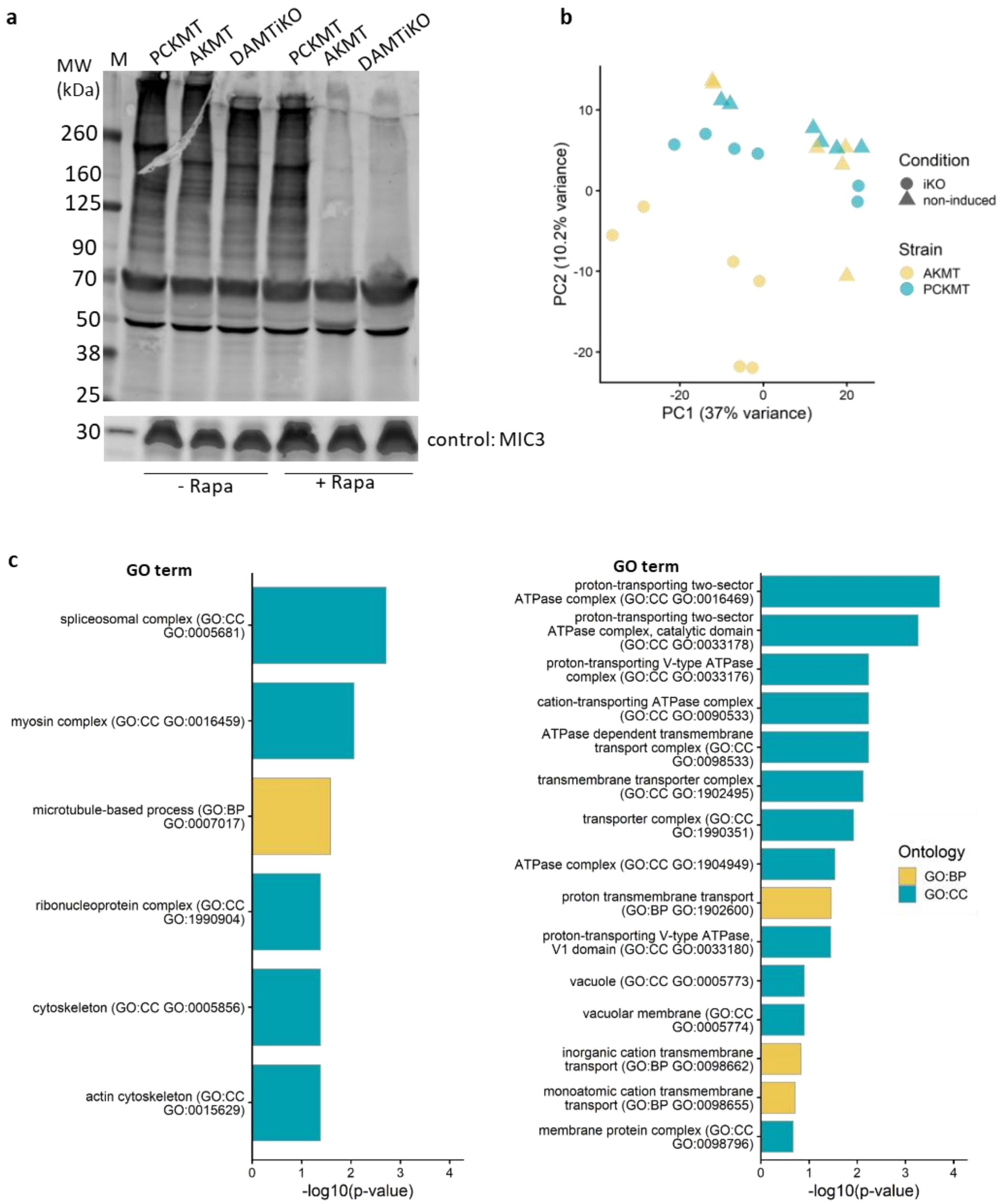

Supplementary Fig. 8 | Validation and analysis of AKMT- and PCKMT-dependent methylation profiles in *T. gondii*.

(a) Western blot analysis of total parasite extracts from the indicated strains—*loxPpckmt-Halo*, *loxPakmt-YFP*, and DAMT-iKO—probed with anti-H4K20me3 antibody to assess global lysine methylation. Equivalent loading was confirmed by probing for GAP45.

(b) Principal component analysis (PCA) of proteomic features from methylome-enriched samples of *T. gondii* PCKMT- and AKMT-knockout strains. Each point represents one sample, n = 3 independent biological replicates per condition, positioned by its scores on PC1 (37% variance) and PC2 (10.2% variance). Colours denote strain (AKMT, yellow; PCKMT, cyan); shapes denote condition (non-induced, circles; iKO, triangles). Tight clustering within groups indicates high reproducibility, while clear separation along PC1/PC2 reflects strain- and induction-specific effects.

(c) GO term enrichment analysis of methylation-enriched proteomes. For AKMT (left graph), significantly decreased proteins in the AKMT-iKO versus wild-type were analysed, revealing enrichment of terms associated with cytoskeletal organisation, pellicle structure, and motility-related

processes. Due to the limited number of significantly altered proteins in PCKMT-iKO (right graph), GO analysis was performed on proteins with a $\log_2$ fold change ≤ –2, yielding no meaningful enrichment. Source data are provided as a Source Data file.

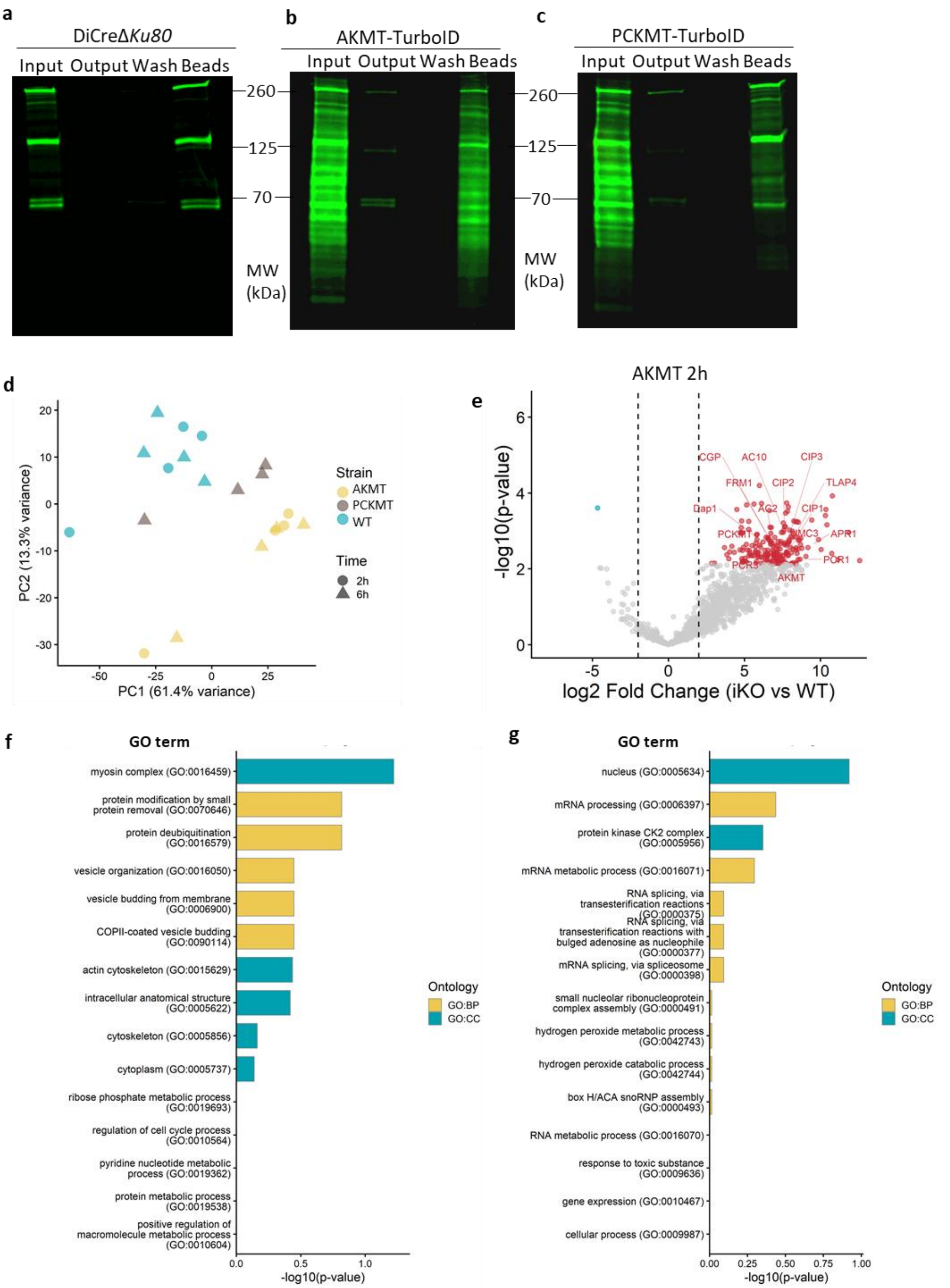

Supplementary Fig. 9 | Validation and analysis of AKMT and PCKMT TurboID proximity-labelling datasets in *T. gondii*.

(a–c) Streptavidin-based detection of biotinylated proteins following TurboID proximity labelling. Parasite lysates were resolved by SDS-PAGE and blotted with streptavidin conjugated to infrared dye (IR-800) for detection on a LI-COR Odyssey system.
(a) Parental DiCreΔKu80 strain (negative control). **(b)** AKMT–TurboID. **(c)** PCKMT–TurboID.
(d) Principal component analysis (PCA) of *T. gondii* TurboID proteomes for PCKMT and AKMT. Each point represents one sample, n = 3 independent biological replicates per condition, positioned by PC1 (37%) and PC2 (10.2%) scores. Colour denotes strain (AKMT, yellow; PCKMT, cyan); shape denotes condition (non-induced, circles; biotin-induced, triangles).

(e) Volcano plot of AKMT–TurboID enrichment after 2 h of biotin induction. Cytoskeletal proteins, including FRM1, DAP1, and PCKMT, are among the enriched interactors. Grey points indicate proteins that did not meet the significance threshold; cyan and red points indicate significant negative and positive $\log_2$ fold changes, respectively.
(f-g) Gene Ontology (GO) enrichment analysis of TurboID datasets. AKMT interactome shows enrichment for cytoskeletal and vesicle organisation terms **(f)**. PCKMT interactome shows enrichment for nuclear and RNA-processing functions **(g).** GO Biological Process (GO: BP) terms are shown in yellow; GO Cellular Component (GO: CC) in blue.
Source data are provided as a Source Data file.

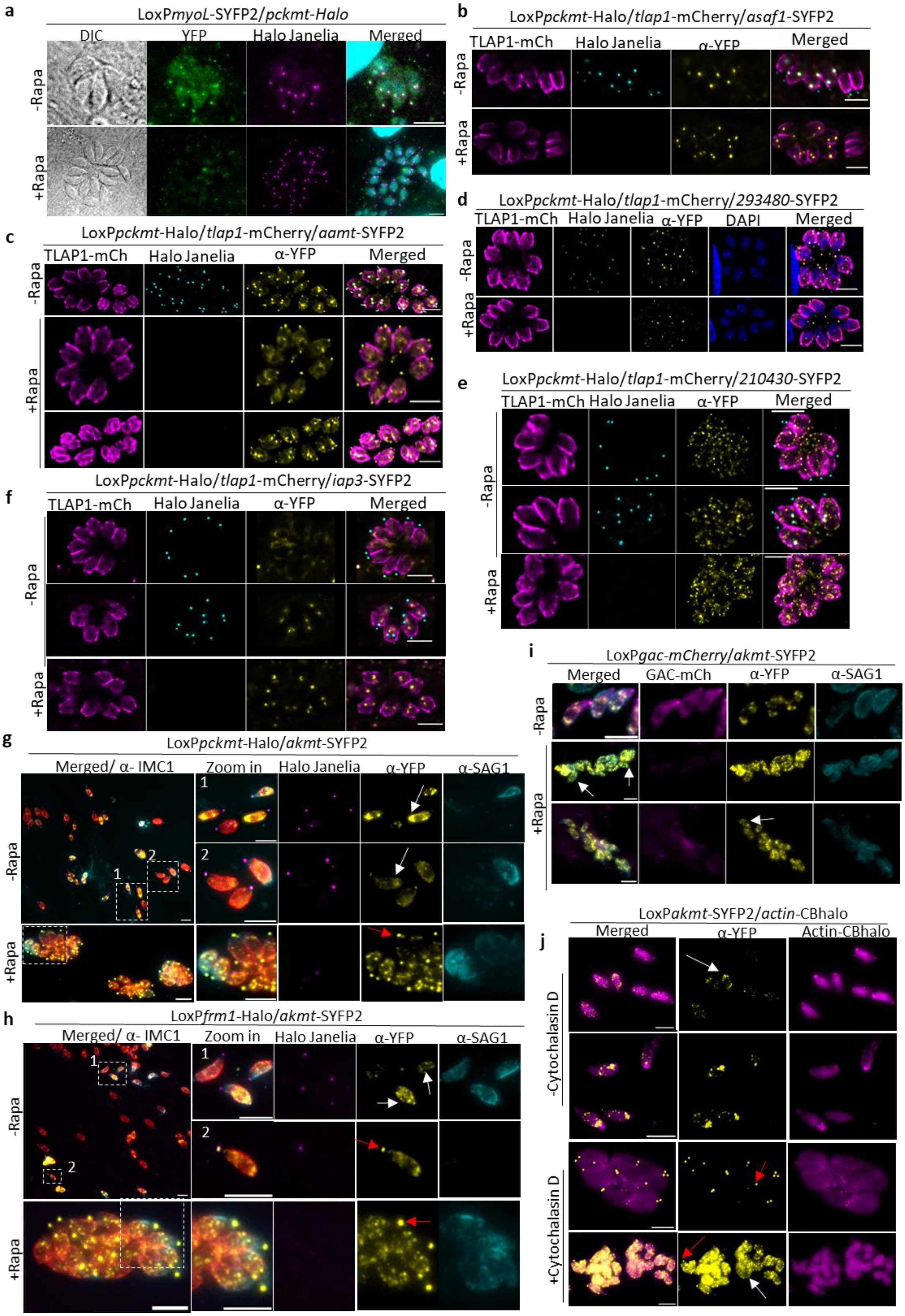

Supplementary Fig. 10 | PCKMT positioning and AKMT relocalisation reveal distinct features of the apical methylation network.

(a) Absence of MyoL does not affect localisation of PCKMT to the conoid. Scale bar, 5 µm.

(b–f) Endogenous tagging of candidate proteins with YFP in the *loxPpckmt* background. Immunofluorescence assays revealed no detectable changes in localisation following PCKMT depletion. Scale bar, 5 µm. **(b)** AAMT. **(c)** TGGT1_212780 (ASAF1). **(d)** TGGT1_238170 (IAP3). **(e)** TGGT1_293480 (MoeA N-terminal region-containing protein). **(f)** TGGT1_210430 (DNAJ domain-containing protein).
(g–j) Overview images of AKMT translocation shown in Fig. 6a–d. Egress was induced with 4 µM CI for 5 min. Anti-SAG1 antibody, shown in cyan, was used to mark extracellular parasites and vacuoles in which the PVM had lysed but parasites had not successfully egressed. IMC, shown in red, marks all parasites. **(g)** AKMT translocation in *loxPpckmt*-Halo parasites. Parasites were pre-induced ±Rapa for at least 54 h. AKMT relocalises to the cytosol, indicated by the white arrow. Scale bar, 5 µm. (1) AKMT sometimes displays a strong cytosolic signal in intracellular parasites. (2) In other cases, AKMT shows a weaker cytosolic signal. **(h)** AKMT translocation in *loxPfrm1*-Halo parasites. (1) Representative image showing differences in AKMT intensity between cytosolic and extracellular parasites, indicated by the white arrow. (2) Representative image showing that, after egress and reinvasion, the newly invaded parasite has AKMT signal at the conoid. Scale bar, 5 µm. **(i)** AKMT translocation in *loxPgac*-mCherry parasites, indicated by the white arrow. Scale bar, 5 µm. **(j)** Parasites were pre-treated with 5 µM cytochalasin D (CytD) for 1 h before egress induction. F-actin was labelled with Chromobody-Halo (CbHalo). In some cases, AKMT relocalisation to the cytosol was observed, indicated by the white arrow, probably reflecting incomplete inhibition of actin dynamics in that vacuole. The red arrow indicates AKMT retained at the conoid. Scale bar, 5 µm.

The images shown are representative of three independent experiments.

Uncropped gels and blots shown in the Supplementary Figures

Supplementary Fig. 1

d

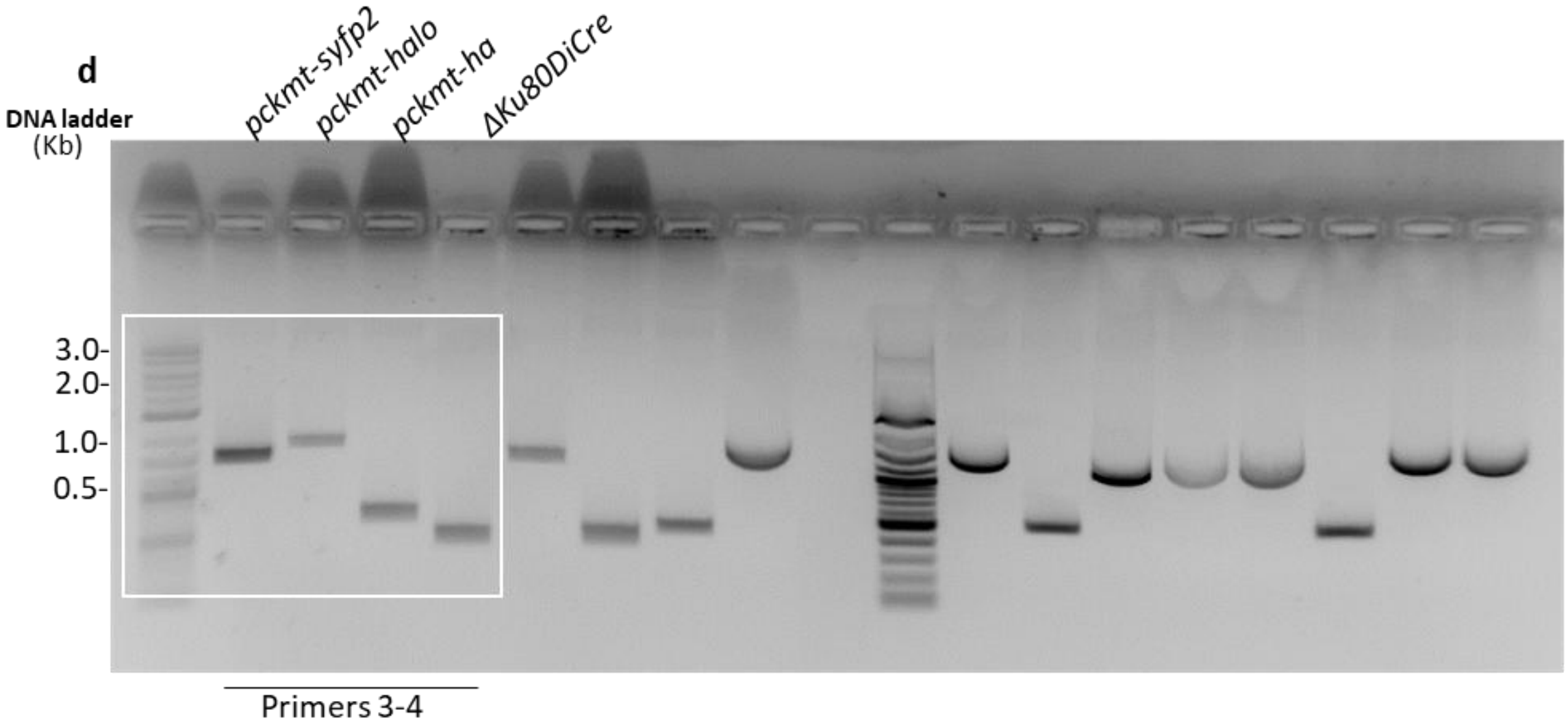

e

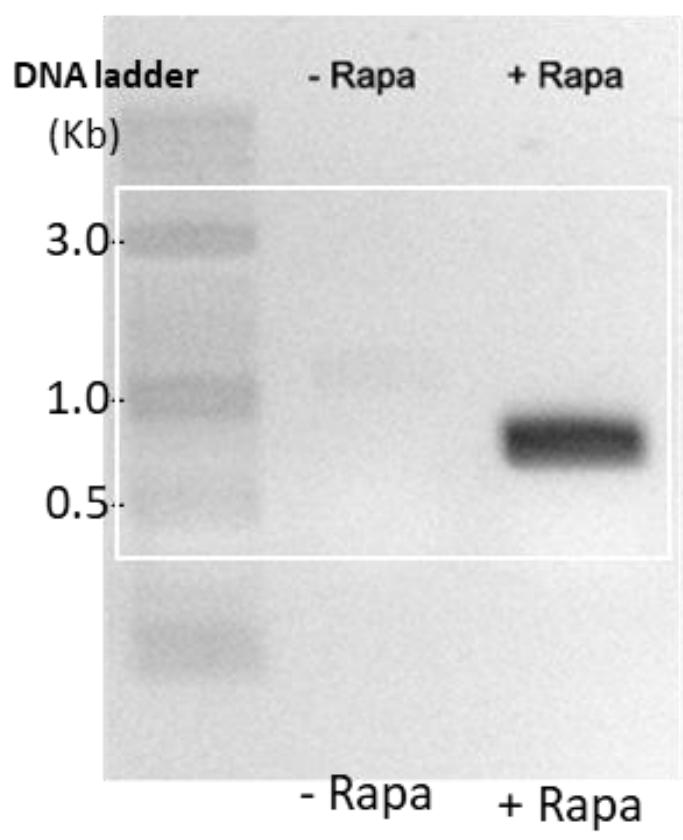

Supplementary Fig. 3

d

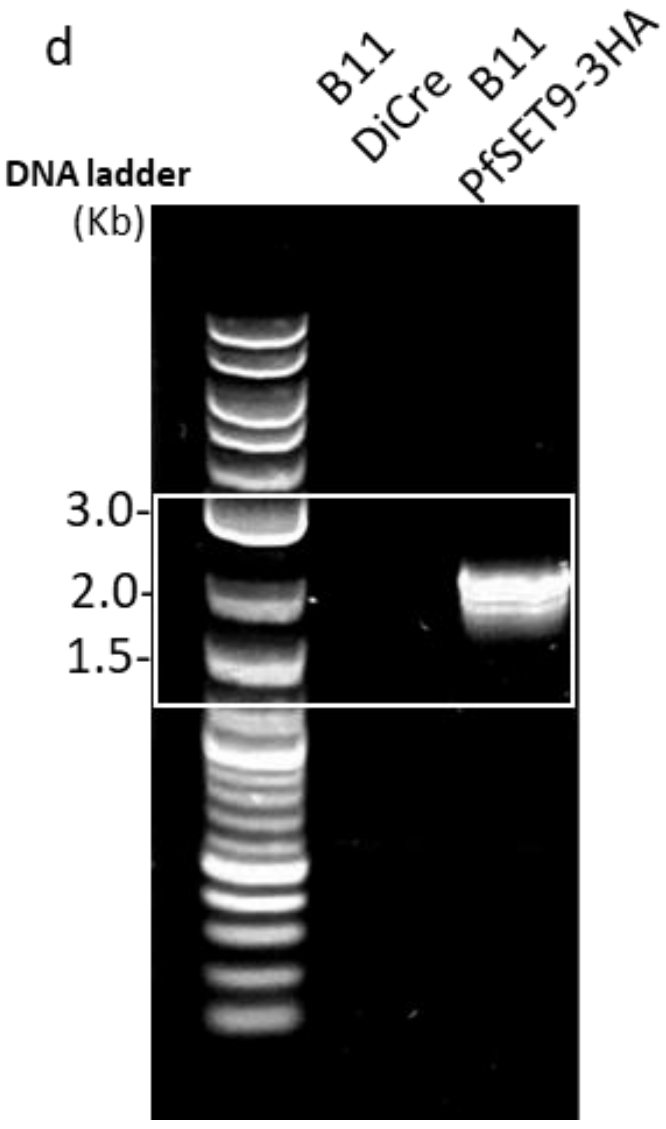

f

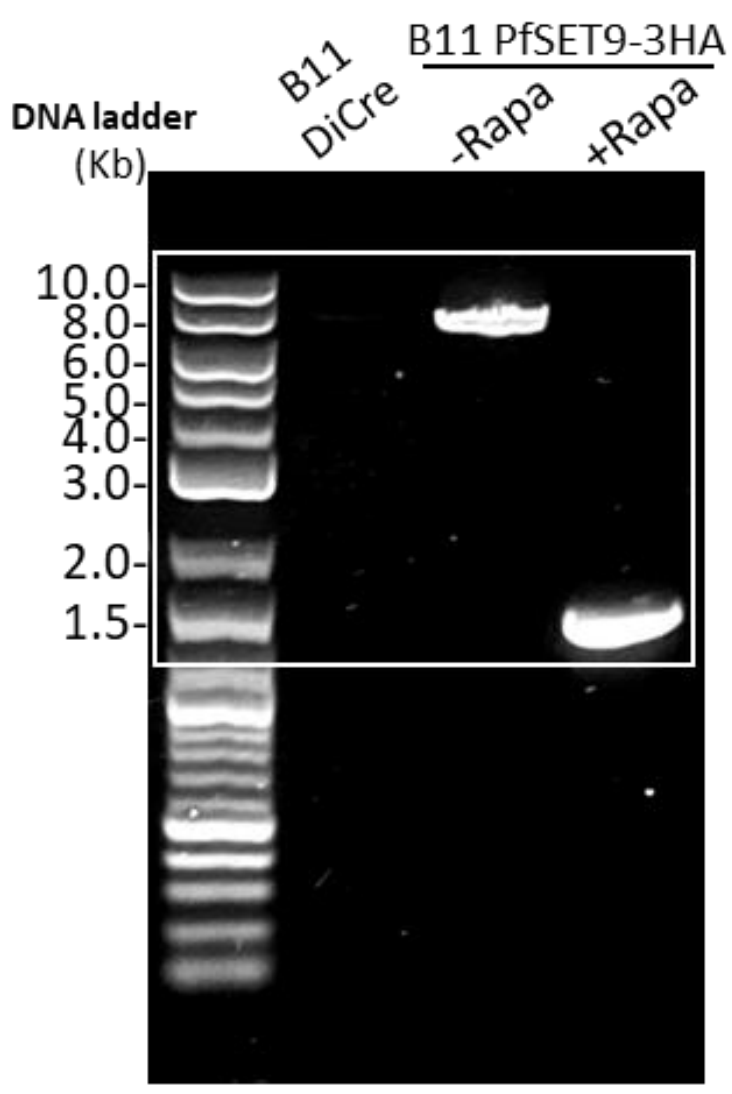

Supplementary Fig. 5

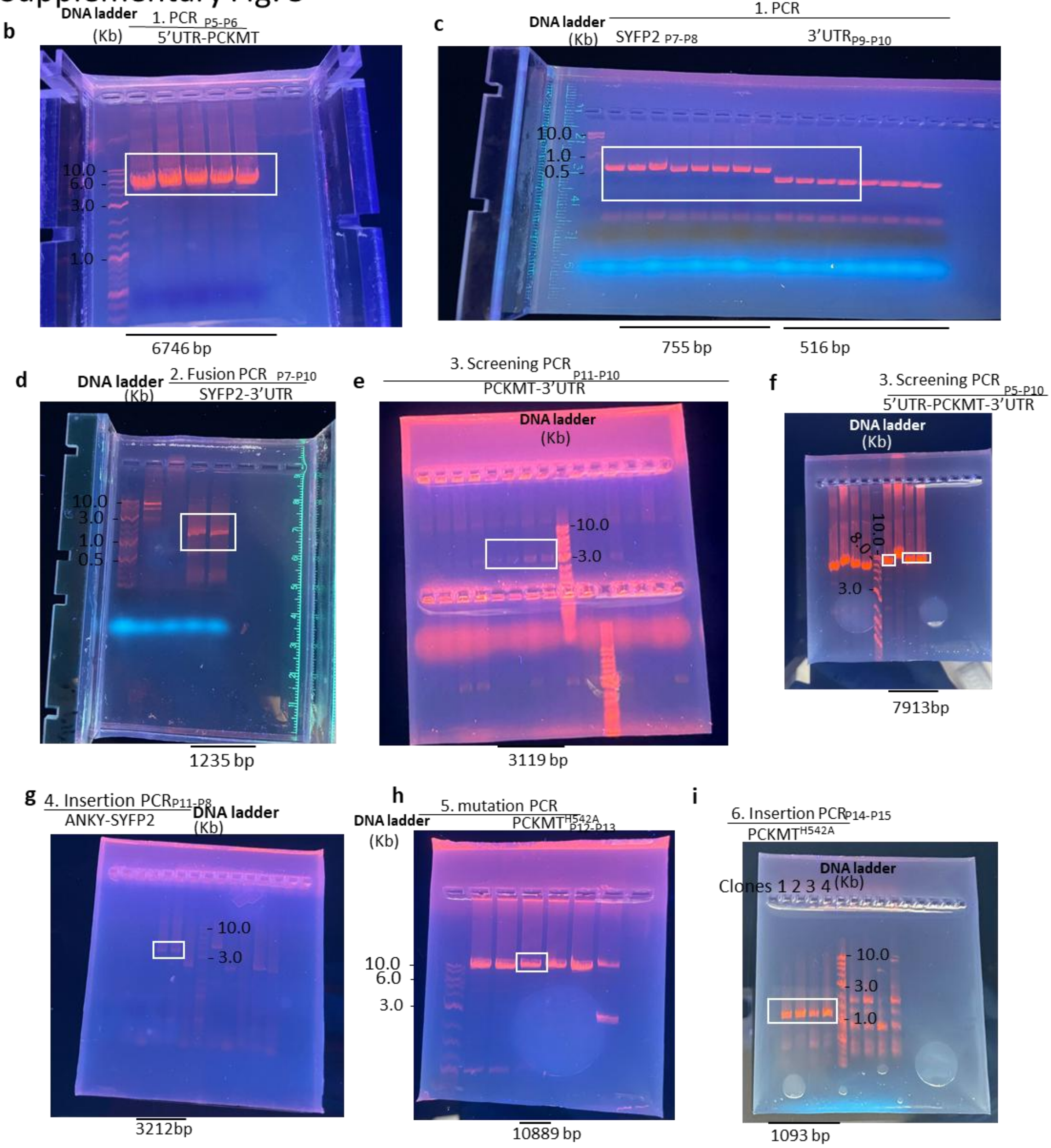

Supplementary Fig. 8

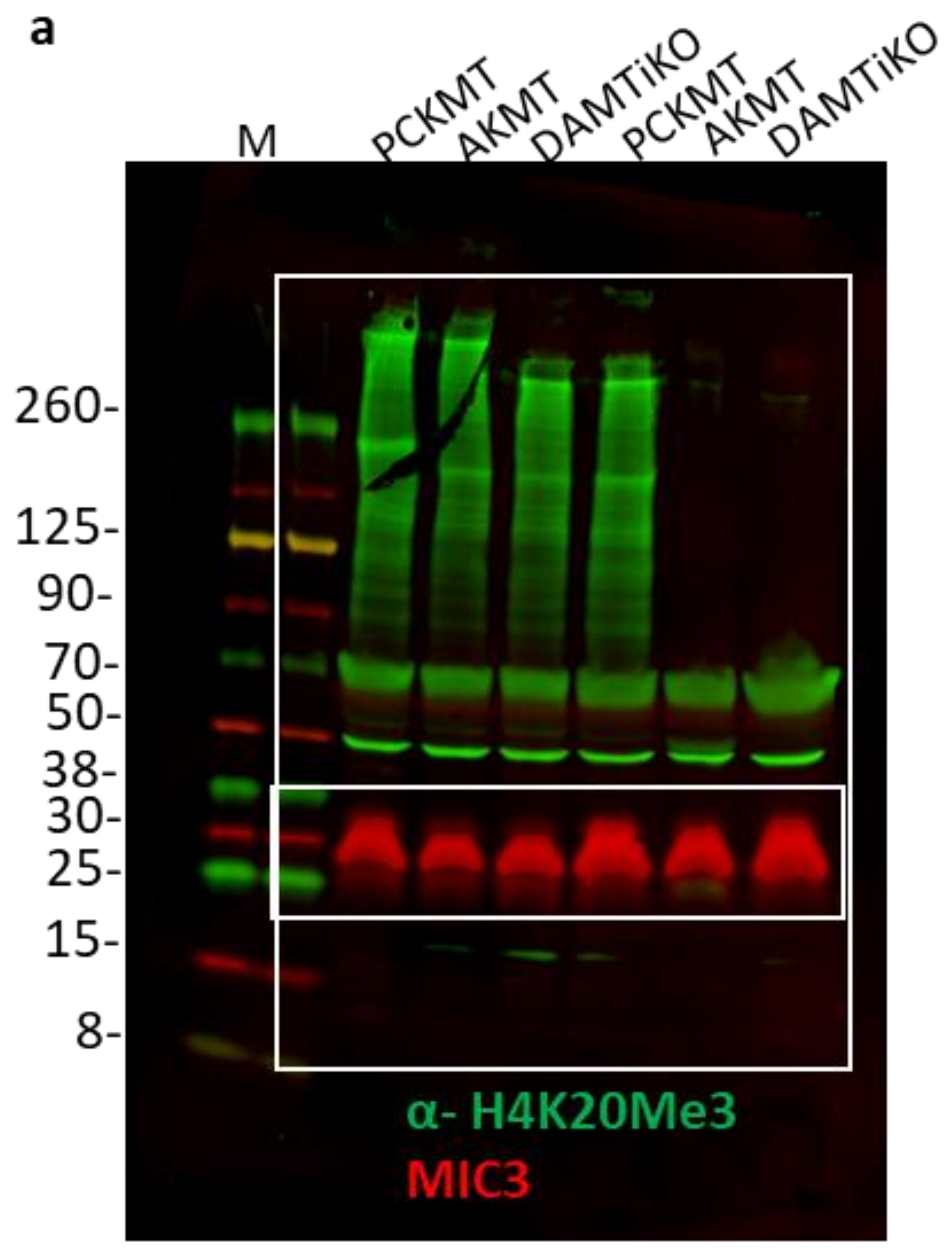

Supplementary Fig. 9

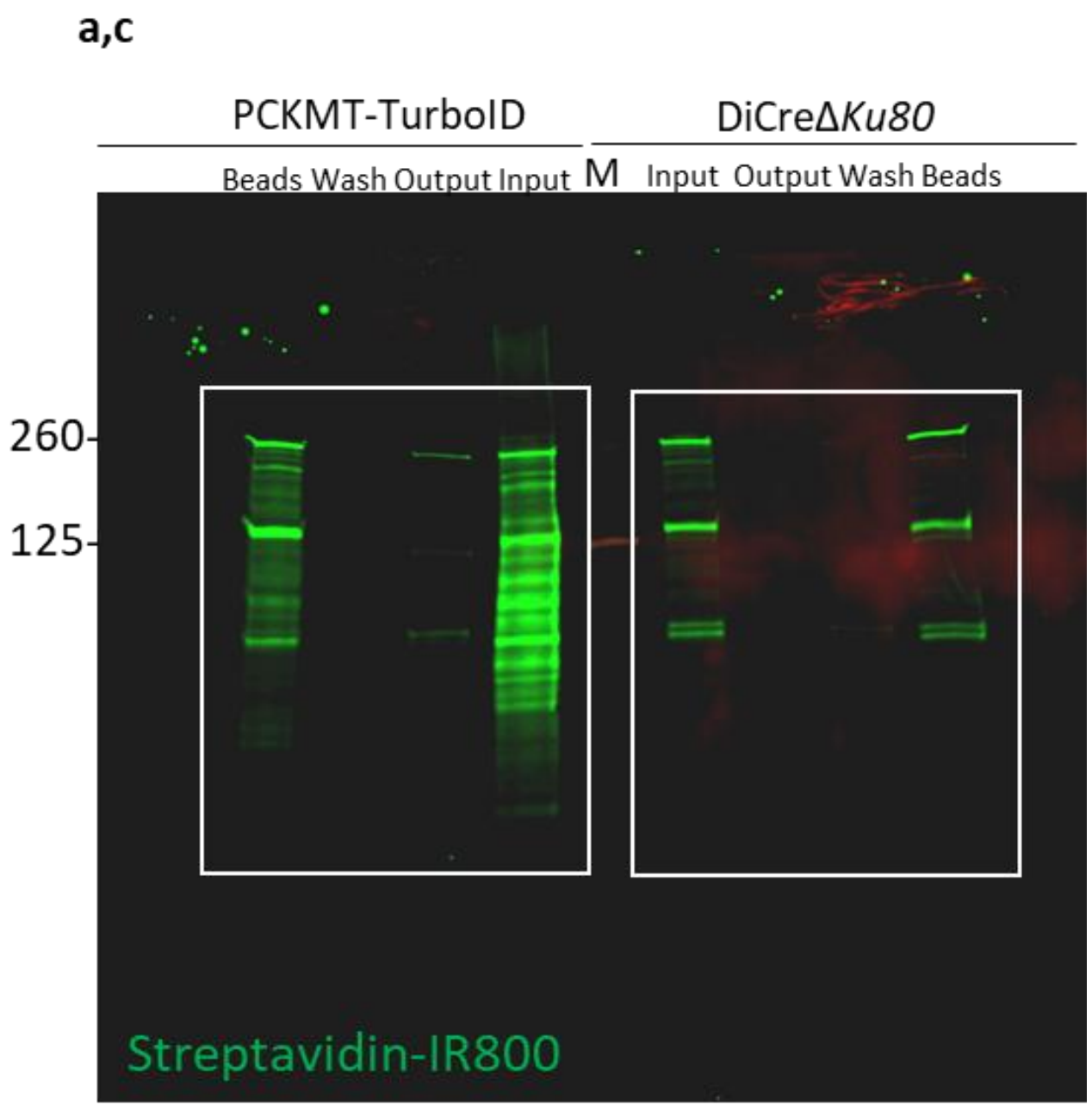

Supplementary Fig. 9

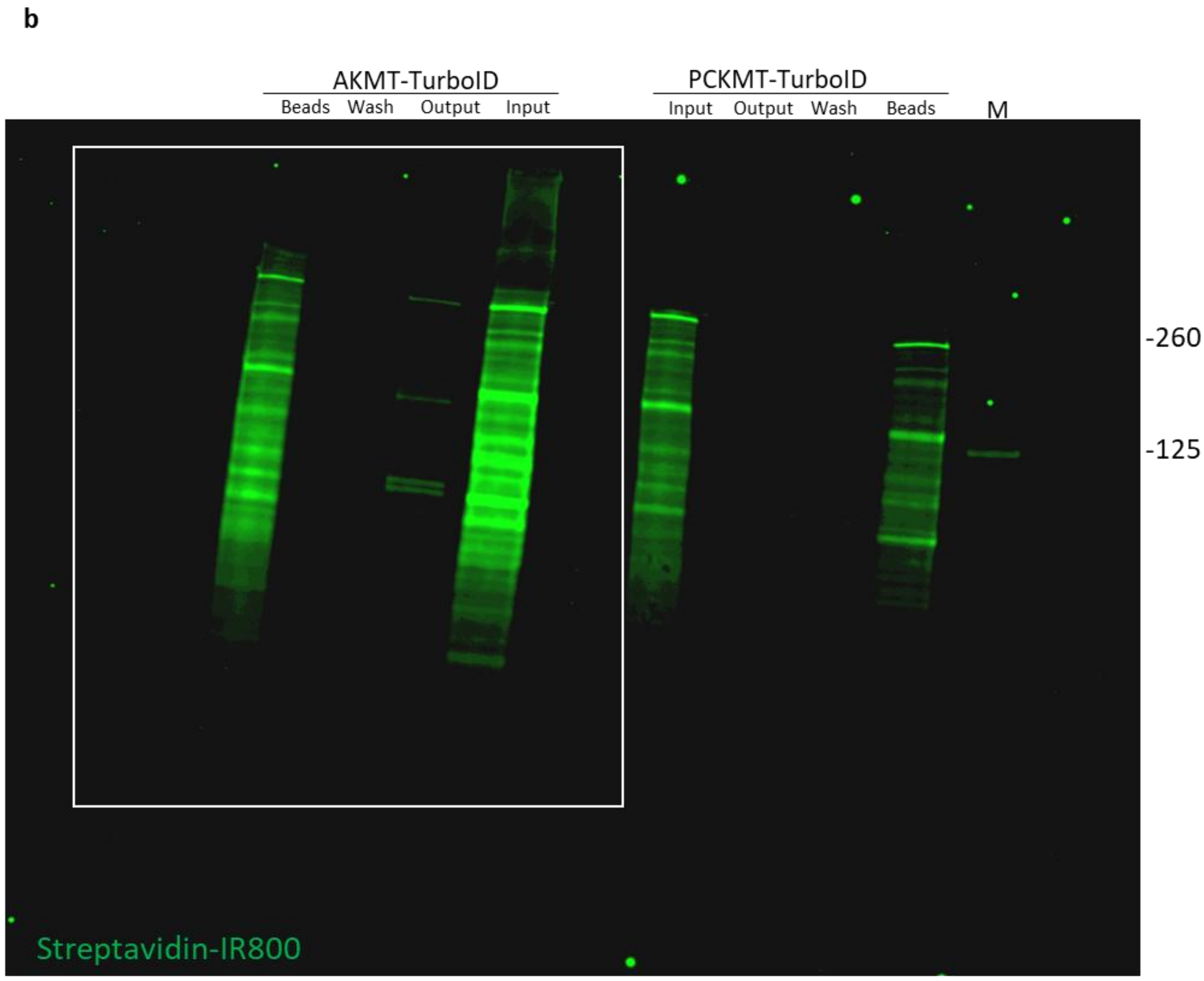

Supplement: Supplementary file 3 [file 41467_2026_77078_MOESM1_ESM.pdf]
